# Supplementary material for: A modular approach for modeling the cell cycle based on functional response curves
Source: PLoS Comput Biol. 2021 Aug 11;17(8):e1009008. doi: 10.1371/journal.pcbi.1009008 (PMC8382204; doi:10.1371/journal.pcbi.1009008)
Supplement: S1 Text — This file contains additional mathematical analysis of the models and the supplemental figures listed below. (PDF) [file pcbi.1009008.s001.pdf]

Supplemental Information

A modular approach for modeling the cell cycle based on functional response curves

The steady-state of a two-variable ultrasensitive negative feedback system without delay is stable

In the main text we presented a two-variable system of the embryonic cell cycle based on an ultrasensitive function:

$$\begin{cases} f([\text{Cdk1}],[\text{APC}]^*) = \dfrac{d[\text{Cdk1}]}{dt} = b_{\text{syn}} - b_{\text{deg}}[\text{Cdk1}]\cdot[\text{APC}]^* \\ g([\text{Cdk1}],[\text{APC}]^*) = \dfrac{d[\text{APC}]^*}{dt} = \dfrac{1}{\epsilon_{\text{apc}}}\left(\dfrac{[\text{Cdk1}]^n}{K^n_{\text{cdk,apc}}+[\text{Cdk1}]^n} - [\text{APC}]^*\right) \end{cases} \tag{1}$$

Here, we will linearize the system to show that the steady-state is stable. The steady-state $([\text{Cdk1}]_{ss}, {[\text{APC}]^*}_{ss})$ of the system can be found by considering the nullclines of the system, i.e. by setting the equations equal to zero:

$$\begin{cases} f([\text{Cdk1}]_{ss},{[\text{APC}]^*}_{ss}) = b_{\text{syn}} - b_{\text{deg}}[\text{Cdk1}]_{ss}\cdot{[\text{APC}]^*}_{ss} = 0 \\ g([\text{Cdk1}]_{ss},{[\text{APC}]^*}_{ss}) = \dfrac{1}{\epsilon_{\text{apc}}}\left(\dfrac{[\text{Cdk1}]^n_{ss}}{K^n_{\text{cdk,apc}}+[\text{Cdk1}]^n_{ss}} - {[\text{APC}]^*}_{ss}\right) = 0 \end{cases} \tag{2}$$

Solving this system of two algebraic equations for $[\text{Cdk1}]_{ss}$ and ${[\text{APC}]^*}_{ss}$ comes down to finding the solution of:

$$b_{\text{deg}}[\text{Cdk1}]^{n+1}_{ss} - b_{\text{syn}}[\text{Cdk1}]^n_{ss} - b_{\text{syn}}K^n_{\text{cdk,apc}} = 0 \tag{3}$$

For $n \geq 4$, Abel's theorem states that the roots of this equation (which is then a polynomial of 5th degree or higher) cannot be expressed as a radical expression in its coefficients [1]. Nevertheless, the number of positive real roots, and therefore the number of steady-states of the system, can be determined via *Descartes' rule of signs*, which states that *the number of its positive roots, with account taken of multiplicities, is equal to the number of changes of sign in the sequence of its coefficients, if the coefficients of the equation are real and all of its roots are also known to be real. If the equation also has complex roots, then this number is equal to or an even number less than the number of the changes in sign* [1]. In our case, all coefficients of Eq. 3 are rate constants and therefore real (positive) numbers. Hence, the sequence of the coefficients, i.e. $b_{\text{deg}}, -b_{\text{syn}}, -b_{\text{syn}}K^n_{\text{cdk,apc}}$, has only one sign change and therefore Eq. 3 has only one unique positive real root.

Although we do not know the exact value of the steady-state of system 1, we can still assess its stability via *linearization* and *local stability analysis*. First, the non-linear system 1 needs to be linearized around its steady-state $([\text{Cdk1}]_{ss}, {[\text{APC}]^*}_{ss})$. Let u and v denote the components of small perturbations around the steady-state:

$$u = [\text{Cdk1}] - [\text{Cdk1}]_{ss} \qquad\qquad v = [\text{APC}]^* - {[\text{APC}]^*}_{ss}$$

The time evolution of the small perturbations u and v can be found from the linearized system [2]:

$$\begin{cases} \dfrac{du}{dt} = \left.\dfrac{\partial f}{\partial[\text{Cdk1}]}\right|_{([\text{Cdk1}]_{ss},[\text{APC}]^*{}_{ss})} \cdot u + \left.\dfrac{\partial f}{\partial[\text{APC}]^*}\right|_{([\text{Cdk1}]_{ss},[\text{APC}]^*{}_{ss})} \cdot v \\ \dfrac{dv}{dt} = \left.\dfrac{\partial g}{\partial[\text{Cdk1}]}\right|_{([\text{Cdk1}]_{ss},[\text{APC}]^*{}_{ss})} \cdot u + \left.\dfrac{\partial g}{\partial[\text{APC}]^*}\right|_{([\text{Cdk1}]_{ss},[\text{APC}]^*{}_{ss})} \cdot v \end{cases} \tag{4}$$

The partial derivatives evaluated at the steady-state ($[\text{Cdk1}]_{ss}$, $[\text{APC}]^*{}_{ss}$) are given by:

$$\begin{aligned} \left.\frac{\partial f}{\partial[\text{Cdk1}]}\right|_{([\text{Cdk1}]_{ss},[\text{APC}]^*{}_{ss})} &= -b_{\text{deg}}[\text{APC}]^*{}_{ss} = -A \\ \left.\frac{\partial f}{\partial[\text{APC}]^*}\right|_{([\text{Cdk1}]_{ss},[\text{APC}]^*{}_{ss})} &= -b_{\text{deg}}[\text{Cdk1}]_{ss} = -B \\ \left.\frac{\partial g}{\partial[\text{Cdk1}]}\right|_{([\text{Cdk1}]_{ss},[\text{APC}]^*{}_{ss})} &= \frac{1}{\epsilon_{\text{apc}}}\left[\frac{nK^n[\text{Cdk1}]_{ss}^{n-1}}{(K^n+[\text{Cdk1}]_{ss}^n)^2}\right] = C \\ \left.\frac{\partial g}{\partial[\text{APC}]^*}\right|_{([\text{Cdk1}]_{ss},[\text{APC}]^*{}_{ss})} &= \frac{-1}{\epsilon_{\text{apc}}} = -D \end{aligned} \tag{5}$$

with the constants A, B, C, and D being real positive numbers. The matrix of partial derivatives is called the *Jacobian matrix* of the system and is here evaluated at the steady-state: $\begin{pmatrix} -A & -B \\ C & -D \end{pmatrix}$

The stability of the steady-state can now be determined via the *eigenvalues* λ of the Jacobian matrix, which can be found by solving the *characteristic equation*: $\begin{vmatrix} -A-\lambda & -B \\ C & -D-\lambda \end{vmatrix} = 0$. Expanding the determinant results in $\lambda^2 + (A+D)\lambda + AD + BC = 0$ and hence

$$\lambda_{\pm} = \frac{-(A+D) \pm \sqrt{(A+D)^2 - 4(AD+BC)}}{2} = \frac{-(A+D) \pm \sqrt{(A-D)^2 - 4BC}}{2} \tag{6}$$

From Eq. 5 and 6 it follows that whenever the eigenvalues are real numbers, i.e. $(A+D)^2 - 4(AD+BC) > 0$, both of them will always be strictly negative. It is readily seen that λ_- is always negative. Furthermore, λ_+ would only become positive whenever $\sqrt{(A+D)^2 - 4(AD+BC)} > A+D$, but since $(A+D)^2 - 4(AD+BC) < (A+D)^2$ it follows that $\sqrt{(A+D)^2 - 4(AD+BC)} < \sqrt{(A+D)^2} = A+D$, thus making it impossible for λ_+ to become positive. Similarly, λ_+ would only be zero whenever $\sqrt{(A+D)^2 - 4(AD+BC)} = A+D$, and thus when $AD+BC = 0$, which again is impossible. Consequently, both λ_+ and λ_- will be negative and the steady-state will always be stable. In case $(A+D)^2 - 4(AD+BC) < 0$, the eigenvalues have an imaginary part and $\lambda_{\pm}$ are complex conjugates. Since $-(A+D)$ is negative (A and D are positive constants), the real part will always be negative and again the steady-state is always stable. This analysis thus shows that the steady-state of the two variable system in Eq. 1 is always stable.

Effect of parameters of ξ on the shape of the S-shaped response and oscillations

The overall **shape of the cubic scaling function** $\xi(x)$ depends on the value of the parameters α and r:

$$\begin{aligned} \xi(x) &= 1 + \alpha \cdot x\,(x-1)\,(x-r) \\ &= 1 + \alpha\left[x^3 - (1+r)x^2 + r \cdot x\right] \qquad \text{with } x \in [0,1] \end{aligned} \tag{7}$$

Parameter α determines the $\xi(x)$ range between the relative maximum and minimum of $\xi(x)$, with increasing α resulting in a larger distance between the extrema. For α being larger than a threshold value

$\alpha_{\max}$, the relative minimum of the scaling function will have a negative $\xi(x)$ coordinate. Here, such conditions were not considered and the parameter α was kept smaller than $\alpha_{\max}$, but always positive to ensure that the maximum of $\xi(x)$ is reached at smaller x-coordinates than the minimum:

$$0 \leq \alpha < \alpha_{\max}$$

The value for $\alpha_{\max}$ can be calculated by determining α when the local minimum of $\xi(x)$ equals zero. This local minimum is found by considering $\xi(x) = 1 + \alpha\left[x^3 - (1+r)x^2 + rx\right]$ and

$$\frac{d\xi}{dx} = \alpha\left[3x^2 - 2(1+r)x + r\right] \tag{8}$$

which is zero if $\alpha = 0$ or

$$x_{\pm} = \frac{2(1+r) \pm \sqrt{4(1+r)^2 - 12r}}{6} = \frac{1 + r \pm \sqrt{(1+r)^2 - 3r}}{3}. \tag{9}$$

As we need the second derivative $\frac{d^2\xi}{dx^2} = \alpha\left[6x - 2(1+r)\right]$ to be positive at the local minimum, we use x_+ to determine $\alpha_{\max}$ from the expression for $\xi(x)$:

$$\xi(x) = 0 \Leftrightarrow \alpha_{\max} = \frac{-1}{x_+^3 - (1+r)x_+^2 + rx_+} = \frac{-1}{x_+(x_+ - r)(x_+ - 1)}$$

Parameter *r* determines the x-coordinate where $\xi(x)$ equals 1 (besides $x = 0$ and $x = 1$ where $\xi(x)$ equals 1 as well). Decreasing r, shifts the extrema of $\xi(x)$ to the left, while larger values of r shift the extrema to higher x-values (see Eq. 9). As the x-coordinates of the extrema decrease, the corresponding $\xi(x)$-coordinates decrease as well (Eq. 7, Fig S2A, S2B, and S2C).

The **overall shape of the S-shaped [APC]* response** with input [Cdk1] (as used in system (i-b) in the main text) is determined by the product of $\xi([\mathrm{APC}]^*)$ and the ultrasensitive Hill function, and thus depends on α, r, the Hill coefficient n and the threshold value $K_{\mathrm{cdk,apc}}$:

$$[\mathrm{Cdk1}] = K_{\mathrm{cdk,apc}} \cdot \xi([\mathrm{APC}]^*) \left(\frac{[\mathrm{APC}]^*}{1 - [\mathrm{APC}]^*}\right)^{1/n} \Rightarrow [\mathrm{APC}]^* = \frac{[\mathrm{Cdk1}]^n}{[K_{\mathrm{cdk,apc}} \cdot \xi([\mathrm{APC}]^*)]^n + [\mathrm{Cdk1}]^n} \tag{10}$$

To assess the **effect of α and r** on the overall shape and position of the S-shaped response, one needs to consider the role of the **Hill coefficient *n*** (Fig S2D, S2E, and S2F). The bigger n, the more the ultrasensitive response approaches a step function and the closer the shape of the inverted S-shaped response will resemble the shape of $\xi([\mathrm{APC}]^*)$: for n going to infinity, the power $1/n$ in Eq. 10 approaches zero and then $[\mathrm{Cdk1}] \approx K_{\mathrm{cdk,apc}} \cdot \xi([\mathrm{APC}]^*)$. Whenever the effect of n cannot be neglected, some discrepancies between the scaling function $\xi([\mathrm{APC}]^*)$ and the S-shaped response exist, e.g. the actual folds in the phase plane lay closer together on the [APC]* axis than the extrema of $\xi([\mathrm{APC}]^*)$ or the S-shaped response curve is not perfectly symmetric around the threshold $K_{\mathrm{cdk,apc}}$, while $\xi([\mathrm{APC}]^*)$ is symmetric around 1 (at least for $r = 0.5$). Consequently, altering α does not only affect the [Cdk1] coordinate of the folds, but can also have an effect on their [APC]* coordinates. For most of the analysis in this study, a value of $n = 15$ was sufficiently high to keep the discrepancies just discussed sufficiently small to allow for correct interpretation of the results. For parameter r, the effect on the shape and position of the S-shaped response curve is more difficult to interpret (even with large values of n) as changing r alters the position of the folds along both axes in the phase plane (Fig S2B and S2C). Therefore, parameter r was always kept constant at 0.5.

In the expression for the S-shaped response curve (see Eq. 10), $\xi([\mathrm{APC}]^*)$ is multiplied by the **threshold value** $\boldsymbol{K_{\mathrm{cdk,apc}}}$, whose effect is twofold (Fig S2G, S2H, and S2I). First, lower or higher values of $K_{\mathrm{cdk,apc}}$ shift the response curve to the left or right in the phase plane respectively, an effect resulting from the product with the first term of $\xi([\mathrm{APC}]^*)$, i.e. 1. Secondly, $K_{\mathrm{cdk,apc}}$ also affects the width of the bistable curve, as it is multiplied by the second term of $\xi([\mathrm{APC}]^*)$ and thus alters the prefactor of the cubic from α to $\alpha K_{\mathrm{cdk,apc}}$ (Fig S2H). One way of keeping the width of the S-shaped response constant while changing the threshold $K_{\mathrm{cdk,apc}}$ is to divide the newly obtained prefactor $\alpha K_{\mathrm{cdk,apc}}$ by $K_{\mathrm{cdk,apc}}$. This strategy was exploited in Fig. 6 of the main text where $K_{\mathrm{cdk,apc}}$ was changed (a similar reasoning holds for changing $K_{\mathrm{cyc,cdk}}$). However, here too the Hill coefficient n needs to be accounted for and the strategy only becomes exact for n approaching infinity:

$$\begin{aligned}
W &= [\mathrm{Cdk1}]_{RF} - [\mathrm{Cdk1}]_{LF} \qquad\qquad \text{with RF = right fold, LF = left fold and } K = K_{\mathrm{cdk,apc}} \\
&= K \cdot \xi([\mathrm{APC}]^*{}_{RF}) \left(\frac{[\mathrm{APC}]^*{}_{RF}}{1-[\mathrm{APC}]^*{}_{RF}} \right)^{1/n} - K \cdot \xi([\mathrm{APC}]^*{}_{LF}) \left(\frac{[\mathrm{APC}]^*{}_{LF}}{1-[\mathrm{APC}]^*{}_{LF}} \right)^{1/n} \\
&= K \Bigg[\left(\frac{[\mathrm{APC}]^*{}_{RF}}{1-[\mathrm{APC}]^*{}_{RF}} \right)^{1/n} + \alpha \left([\mathrm{APC}]^{*3}_{RF} - (1+r)[\mathrm{APC}]^{*2}_{RF} + r[\mathrm{APC}]^*{}_{RF} \right) \left(\frac{[\mathrm{APC}]^*{}_{RF}}{1-[\mathrm{APC}]^*{}_{RF}} \right)^{1/n} \\
&\quad - \left(\frac{[\mathrm{APC}]^*{}_{LF}}{1-[\mathrm{APC}]^*{}_{LF}} \right)^{1/n} - \alpha \left([\mathrm{APC}]^{*3}_{LF} - (1+r)[\mathrm{APC}]^{*2}_{LF} + r[\mathrm{APC}]^*{}_{LF} \right) \left(\frac{[\mathrm{APC}]^*{}_{LF}}{1-[\mathrm{APC}]^*{}_{LF}} \right)^{1/n} \Bigg]
\end{aligned} \tag{11}$$

This expression for the width W of the S-shaped response can indeed become independent of K if we replace α by α/K, but only if $\left(\frac{[\mathrm{APC}]^*{}_{RF}}{1-[\mathrm{APC}]^*{}_{RF}} \right)^{1/n} - \left(\frac{[\mathrm{APC}]^*{}_{LF}}{1-[\mathrm{APC}]^*{}_{LF}} \right)^{1/n} = 0$, i.e. for n being large. The S-shaped $[\mathrm{APC}]^*$ response curve will thus become wider for larger values of $K_{\mathrm{cdk,apc}}$ if the prefactor α is not divided by $K_{\mathrm{cdk,apc}}$ (Fig S2H).

Calculating the width of the S-shaped region

In the main text, we determined the width of the S-shaped response curve $y = \frac{x^n}{\xi(y)^n + x^n}$ by directly writing x as function of y and then calculating the roots of the derivative $\frac{dx}{dy}$. For the switch which has $x = [\mathrm{CycB}]^*$ and $y = [\mathrm{Cdk1}]^*/d[\mathrm{CycB}]^*$, the width of the region needs to be calculated in the $[\mathrm{CycB}]^*, [\mathrm{Cdk1}]^*$ - plane. If we write $y = z/x$, we can write the steady-state response as

$$z = \frac{x^n}{\xi(z/x)^n + x^n} x. \tag{12}$$

Here z is proportional to $[\mathrm{Cdk1}]^*$ and x corresponds to $[\mathrm{CycB}]^*$. We are thus interested in calculating the width of the S-shaped region in the (x, z) plane. As before, this is the region in between the fold points. At those points, $\frac{dx}{dz} = 0$.

In this case, it is not straightforward to calculate an explicit expression for $\frac{dx}{dz}$ as function of z. In fact, x is not necessarily a single-valued function of z for this switch. However, we can calculate the roots of $\frac{dx}{dz}$ without such an explicit formula of $\frac{dx}{dz}$ as function of z.

First, we rewrite Eq. (12) as

$$x = \xi(z/x) \left(\frac{z/x}{1 - z/x} \right)^{1/n} = \xi(y) \left(\frac{y}{1-y} \right)^{1/n}. \tag{13}$$

Here we use $y = z/x$. In the right-hand side, x still appears. This formula is thus still an *implicit* definition of x as function of z. We will now take the derivative of the above expression with respect to z. It

is important to note that we consider x as function of z, such that $y = z/x(z)$ and

$$\frac{dy}{dz} = \frac{d}{dz}\left(z/x(z)\right) = (x(z) - z\frac{dx}{dz})/x(z)^2.$$

Taking the derivative with respect to z of Eq. (13) yields, using the chain rule

$$\frac{dx}{dz} = \frac{d}{dy}\left[\xi(y)\left(\frac{y}{1-y}\right)^{1/n}\right]_{y=z/x} \times \frac{dy}{dz}, \tag{14}$$

where $\frac{dy}{dz}$ is given by the formula above. This means that Eq. (14) does not give us an explicit expression of $\frac{dx}{dz}$ as function of z. However, this is not needed since we are only interested in the values of x and z where the expression is zero. These can be computed by calculating the values of y where

$$\frac{d}{dy}\left[\xi(y)\left(\frac{y}{1-y}\right)^{1/n}\right] = 0,$$

which is explained in the Methods section of the main text. By solving this, we obtain the two values for y at the extrema. Substituting these into Eq. (13) yields the x-values. Subtracting the x-values of the extrema gives the width of the S-shaped region.

Nullclines of the three-dimensional system

The three-dimensional system was given by:

$$\begin{cases} \dfrac{d[\text{CycB}]^*}{dt} = \dfrac{c}{d} - [\text{CycB}]^* \cdot [\text{APC}]^* \\ \dfrac{d[\text{Cdk1}]^*}{dt} = \dfrac{1}{\epsilon_{\text{cdk}} \cdot b_{\text{deg}}}\left(d\dfrac{[\text{CycB}]^{*n+1}}{\xi\left({[\text{Cdk1}]^*}/{d\cdot[\text{CycB}]^*}\right)^n + [\text{CycB}]^{*n}} - [\text{Cdk1}]^*\right) \\ \dfrac{d[\text{APC}]^*}{dt} = \dfrac{1}{\epsilon_{\text{apc}} \cdot b_{\text{deg}}}\left(\dfrac{[\text{Cdk1}]^{*n}}{\xi([\text{APC}]^*)^n + [\text{Cdk1}]^{*n}} - [\text{APC}]^*\right) \end{cases}$$

The nullclines thus being:

$$\begin{aligned} \frac{d[\text{CycB}]^*}{dt} &= 0 \Leftrightarrow [\text{APC}]^* = \frac{c}{d\cdot[\text{CycB}]^*} \\ \frac{d[\text{Cdk1}]^*}{dt} &= 0 \Leftrightarrow \frac{[\text{CycB}]^{*n}}{\xi(Y)^n + [\text{CycB}]^{*n}} = Y \qquad \text{with } Y = \frac{[\text{Cdk1}]^*}{d\cdot[\text{CycB}]^*} \\ &\Leftrightarrow [\text{CycB}]^* = \xi(Y)\cdot\sqrt[n]{\frac{Y}{1-Y}} \\ \frac{d[\text{APC}]^*}{dt} &= 0 \Leftrightarrow [\text{Cdk1}]^* = \xi([\text{APC}]^*)\cdot\sqrt[n]{\frac{[\text{APC}]^*}{1-[\text{APC}]^*}} \end{aligned}$$

Converting $[\text{CycB}]^*$ and $[\text{Cdk1}]^*$ to original dimensions gives:

$$\begin{cases} [\text{CycB}] = K_{\text{cyc,cdk}} \cdot [\text{CycB}]^* \\ [\text{Cdk1}] = K_{\text{cdk,apc}} \cdot [\text{Cdk1}]^* = K_{\text{cdk,apc}} \cdot Y \cdot d \cdot [\text{CycB}]^* = Y \cdot [\text{CycB}]^* \cdot K_{\text{cyc,cdk}} \end{cases}$$

References

1. Aleksandrov AD, Kolmogorov AN, Lavrent'ev MA. Mathematics: Its Content, Methods and Meaning. Dover Publications; 2012.
2. Strogatz SH. Nonlinear Dynamics and Chaos. 2nd ed. Westview Press; 2015.

Supplemental Figures

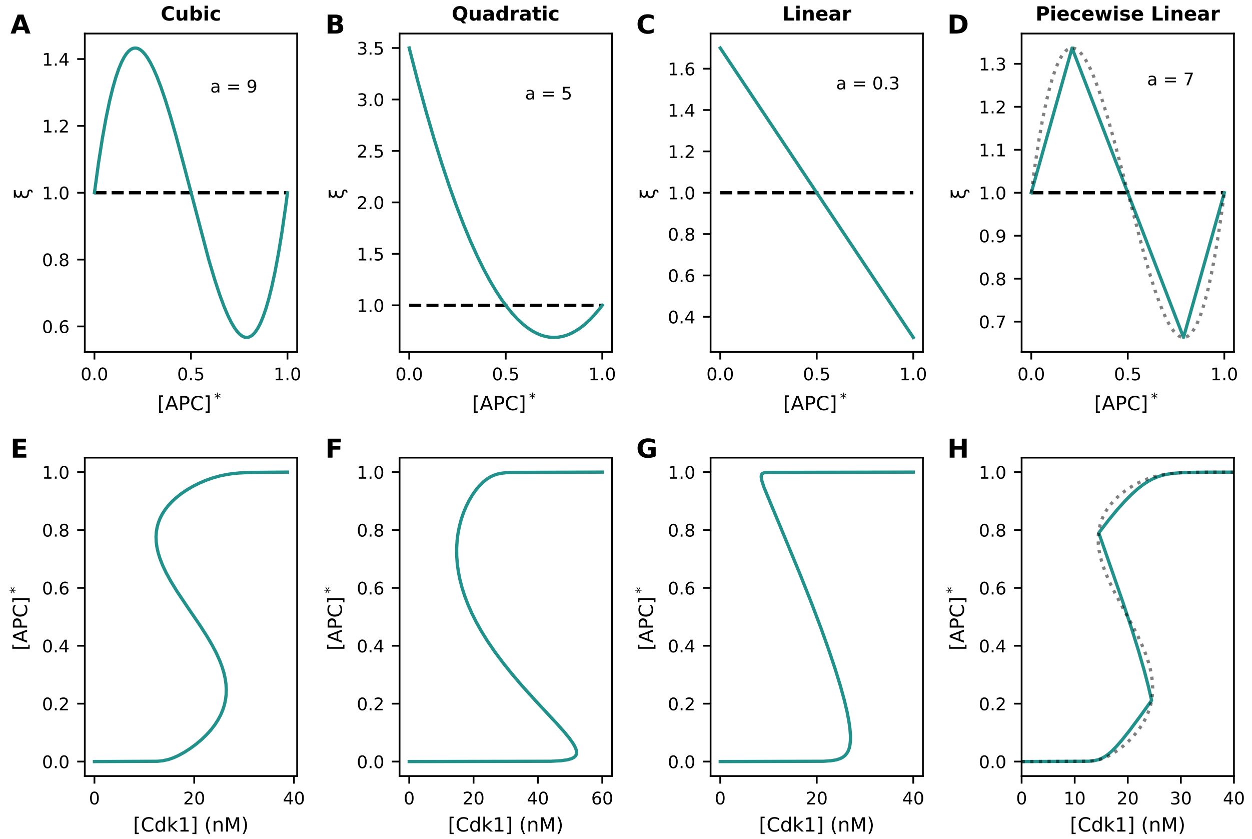

S1 Fig. Alternative definitions of the scaling function ξ. (A-D) Except from the cubic definition of ξ in the main text, alternative definitions can be used. An example of a quadratic function would be

$$\xi([\mathrm{APC}]^*) = 1 + \alpha_{\mathrm{apc}}\left([\mathrm{APC}]^* - 1\right)\left([\mathrm{APC}]^* - r\right)$$

For a linear function, one possible definition would be

$$\xi([\mathrm{APC}]^*) = \frac{1-\alpha_{\mathrm{apc}}}{r-1}\left([\mathrm{APC}]^* - 1\right) + \alpha_{\mathrm{apc}}$$

The equation for the piecewise linear approximation is given in the Methods section of the main text. In each case, $r = 0.5$. **(E-H)** Corresponding response curves obtained by multiplying a Hill function ($K = 20$, $n = 15$) with the scaling functions in panels A-D.

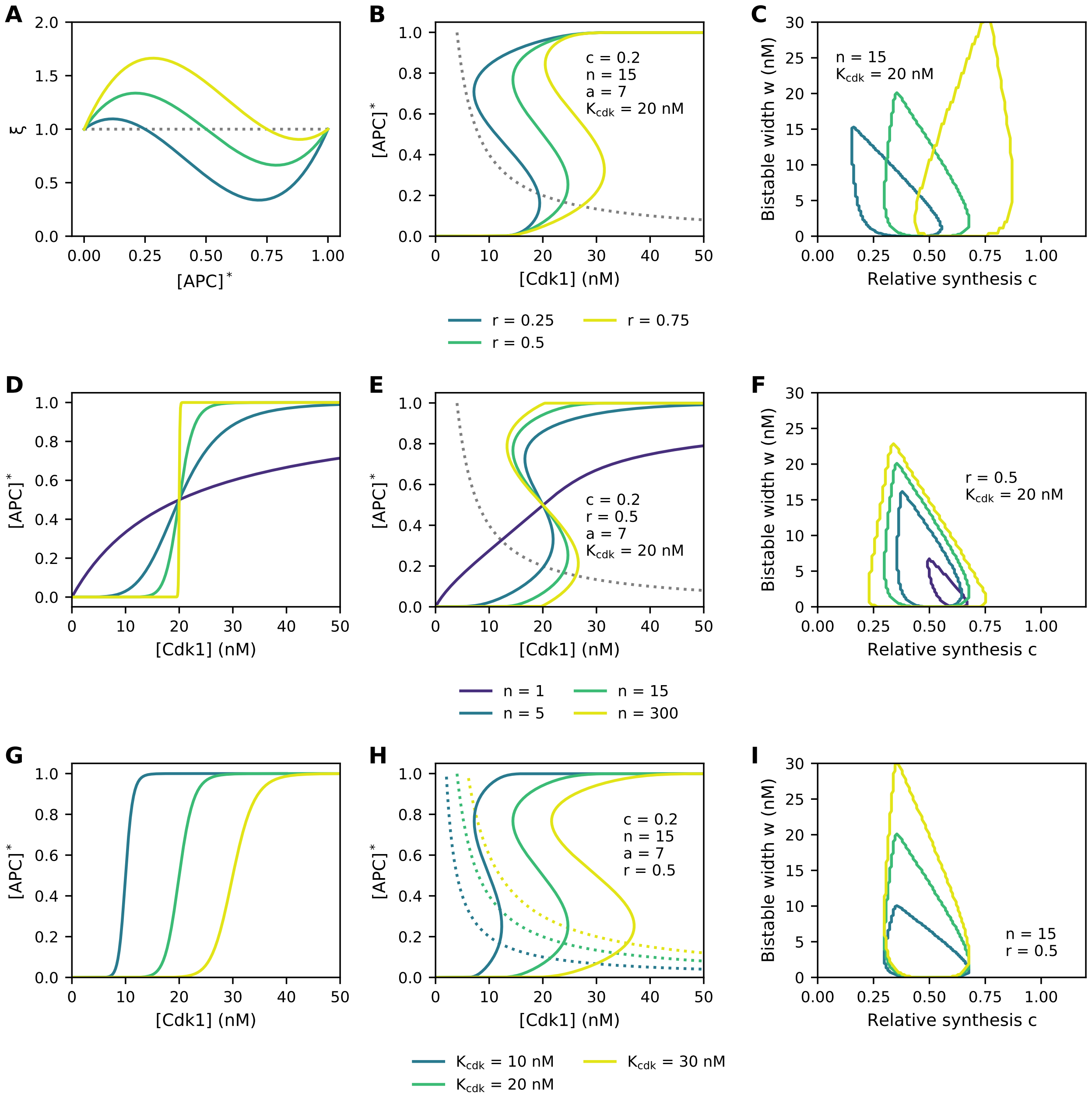

S2 Fig. Effect of parameters on the scaling function and system response. (A-C) Effect of parameter r on the scaling function ξ (A), S-shaped response curve in the phase plane (B) and oscillatory region in the parameter space (C). **(D-F)** Effect of the Hill coefficient n on the original ultrasensitive response (D), the derived S-shaped response curve in the phase plane (E) and oscillatory region in the parameter space (F). **(G-I)** Effect of threshold K on the original ultrasensitive response (G), S-shaped response curve in the phase plane (H) and oscillatory region in the parameter space (I).

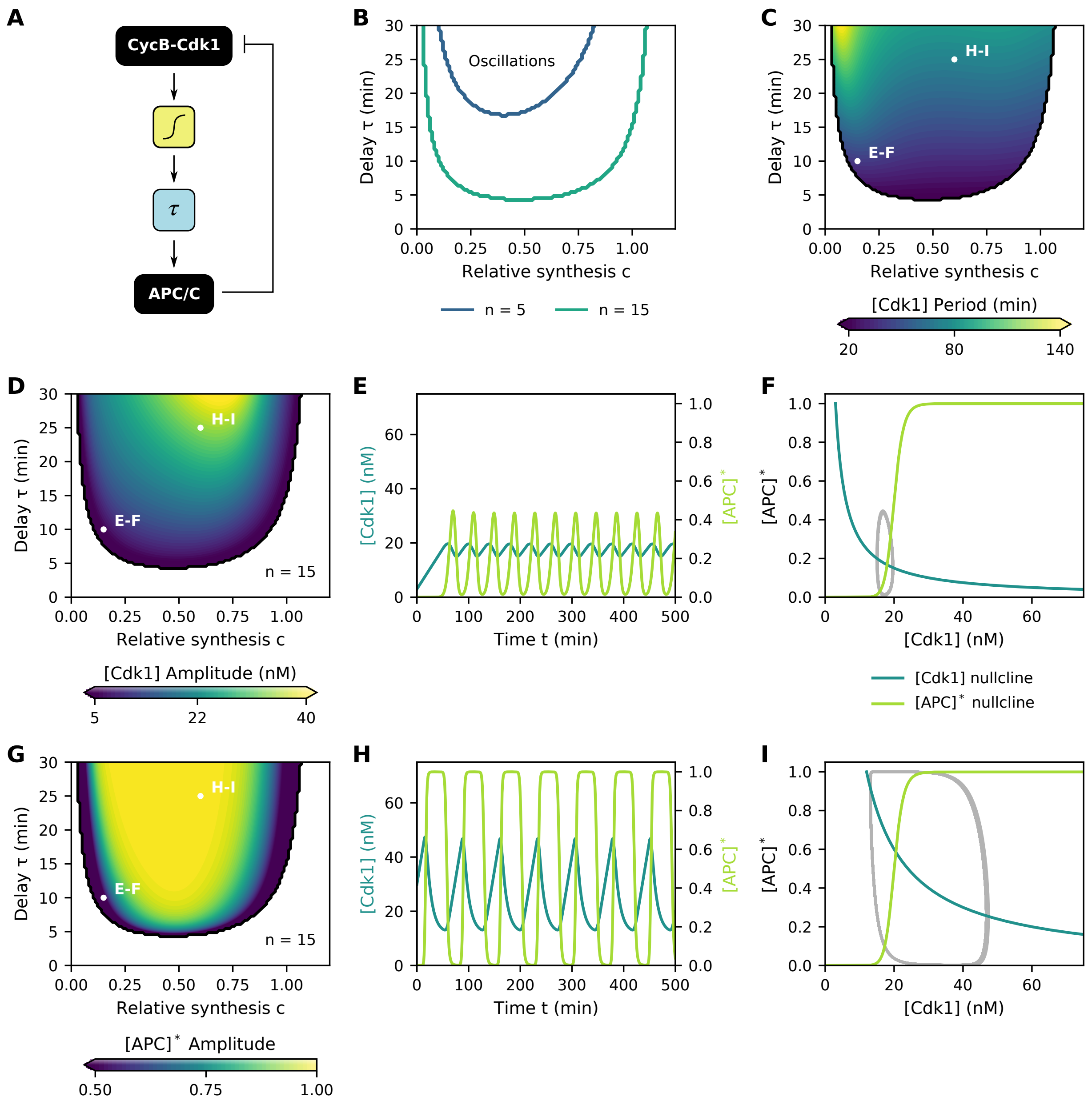

S3 Fig. Oscillations for a time delayed, ultrasensitive cell cycle model. (A) Block diagram of the ultrasensitive, delayed negative feedback network. **(B)** Oscillatory regions for different values of the Hill coefficient n. **(C)** Period of the [Cdk1] oscillations as a function of the relative synthesis rate c and time delay. **(D,G)** [Cdk1] and $[APC]^*$ amplitudes as a function of the relative synthesis rate c and time delay. **(E,F,H,I)** Time traces and phase planes for indicated parameter values.

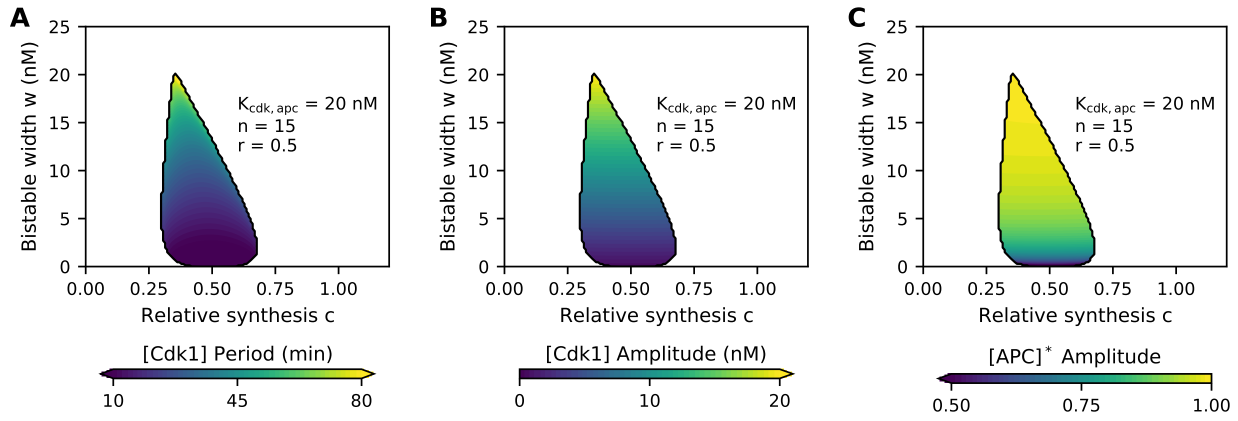

S4 Fig. Oscillation period and amplitude for the S-shaped module. **(A)** [Cdk1] period as a function of the relative synthesis c and the width of the S-shaped region. **(B)** [Cdk1] amplitude as a function of the relative synthesis c and the width of the S-shaped region. **(C)** [APC]* amplitude as a function of the relative synthesis c and the width of the S-shaped region.

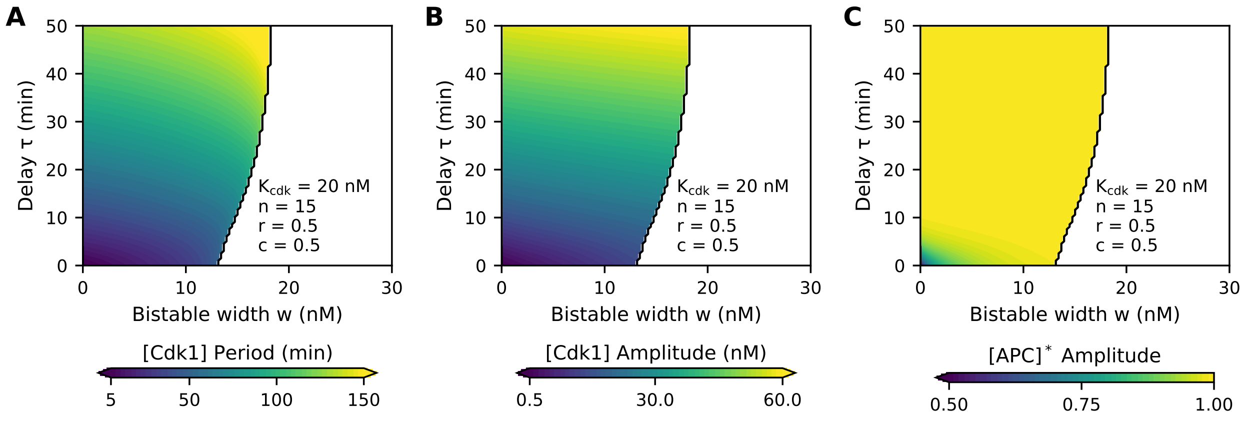

S5 Fig. Oscillation period and amplitude for the delayed S-shaped module. **(A)** [Cdk1] period as a function of the width of the S-shaped region and delay. **(B)** [Cdk1] amplitude as a function of the width of the S-shaped region and delay. **(C)** [APC]* amplitude as a function of the width of the S-shaped region and delay.

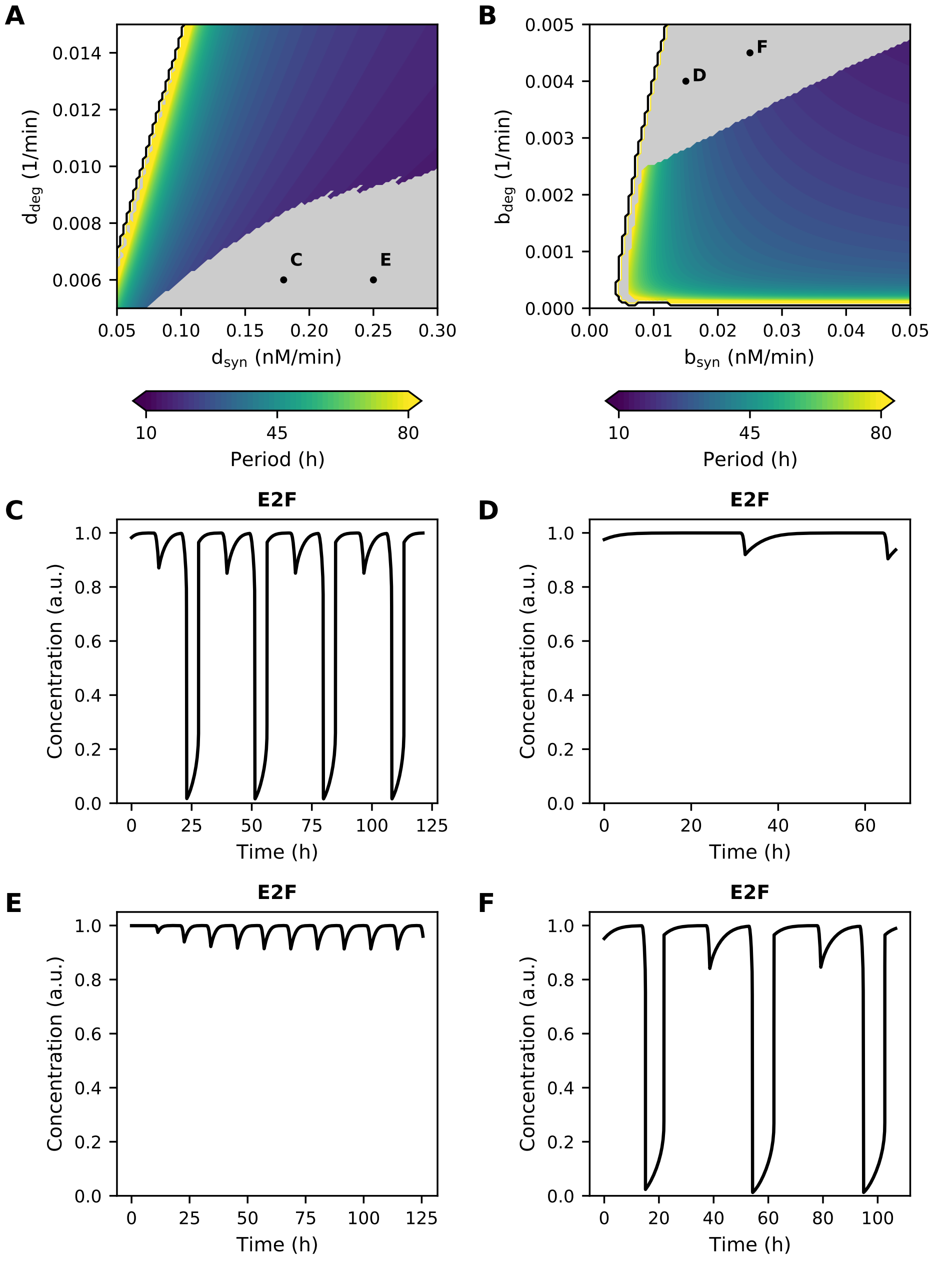

S6 Fig. Irregular cell cycle oscillations in a chain of bistable switches. In Fig 7 in the main text we indicated grey regions in parameter space for which irregular oscillations were observed. Here, we show time traces of $[\text{E2F}]^*$ for such irregular patterns.

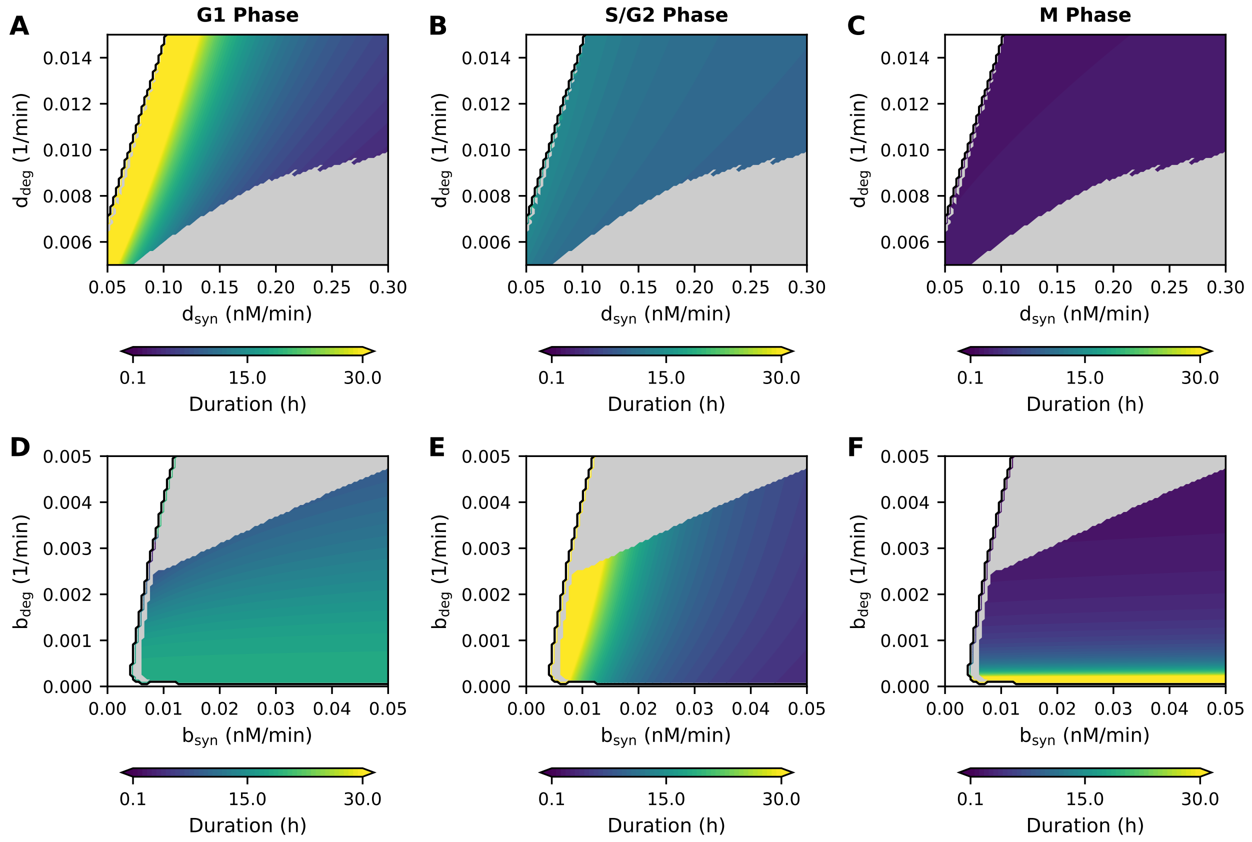

S7 Fig. Effect of synthesis and degradation rates of CycD and CycB on the duration of different cell cycle phases. In Fig 7 in the main text we showed the effect of changing synthesis and degradation rates on the overall length of the cell cycle. Here, we separate the effects on the different cell cycle phases. White areas represent regions where no oscillations can be observed, while for the grey areas irregular oscillations exist.

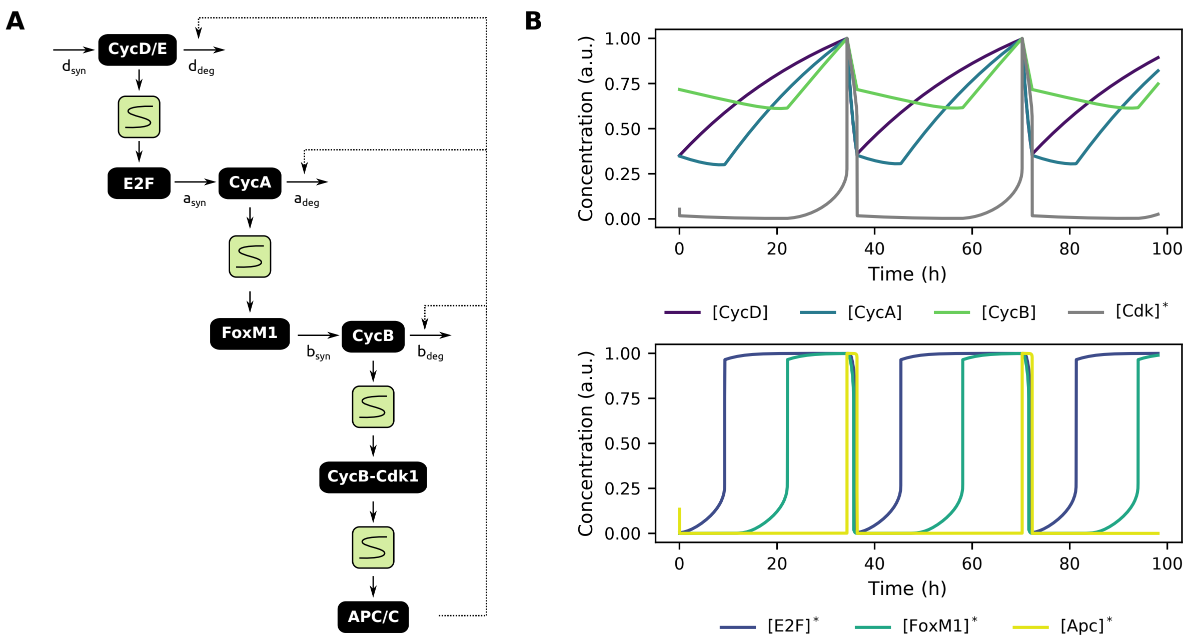

S8 Fig. Extension of the cell cycle model by additional switches. In Fig 7 in the main text we represented the cell cycle as a chain of three bistable switches. Here, we extended this model by including the hypothetical switch of FoxM1 activity with respect to CycA levels.

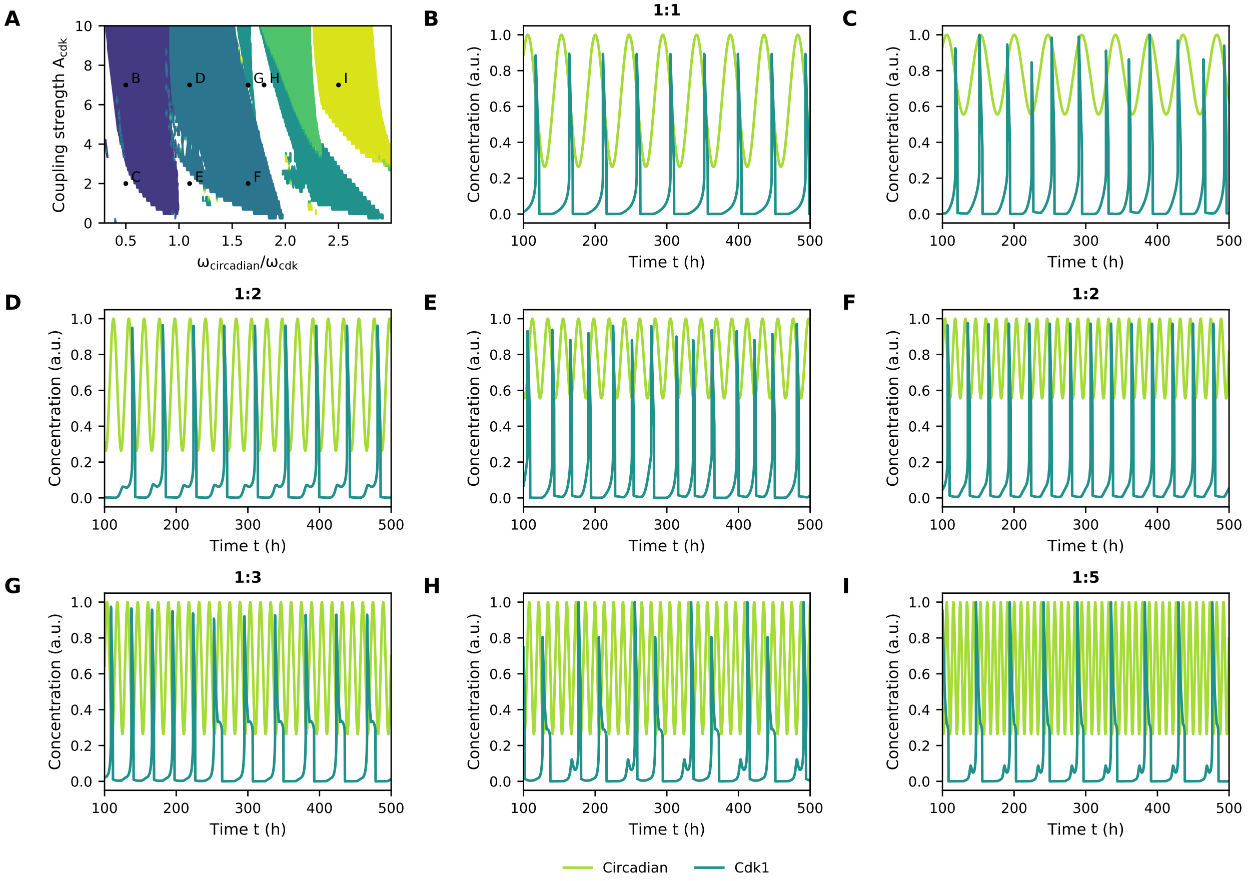

S9 Fig. Phase locking between the circadian clock and the cell cycle. Time traces showing the absence or presence of p:q phase locking (with p and q in [1,2,3,4,5]) between the cell cycle and the circadian clock for several parameter combinations. Related to the Arnold tongues shown in Fig 8.
