# Supplementary figures and images for: A modular approach for modeling the cell cycle based on functional response curves
Source: PLoS Comput Biol. 2021 Aug 11;17(8):e1009008. doi: 10.1371/journal.pcbi.1009008 (PMC8382204; doi:10.1371/journal.pcbi.1009008)

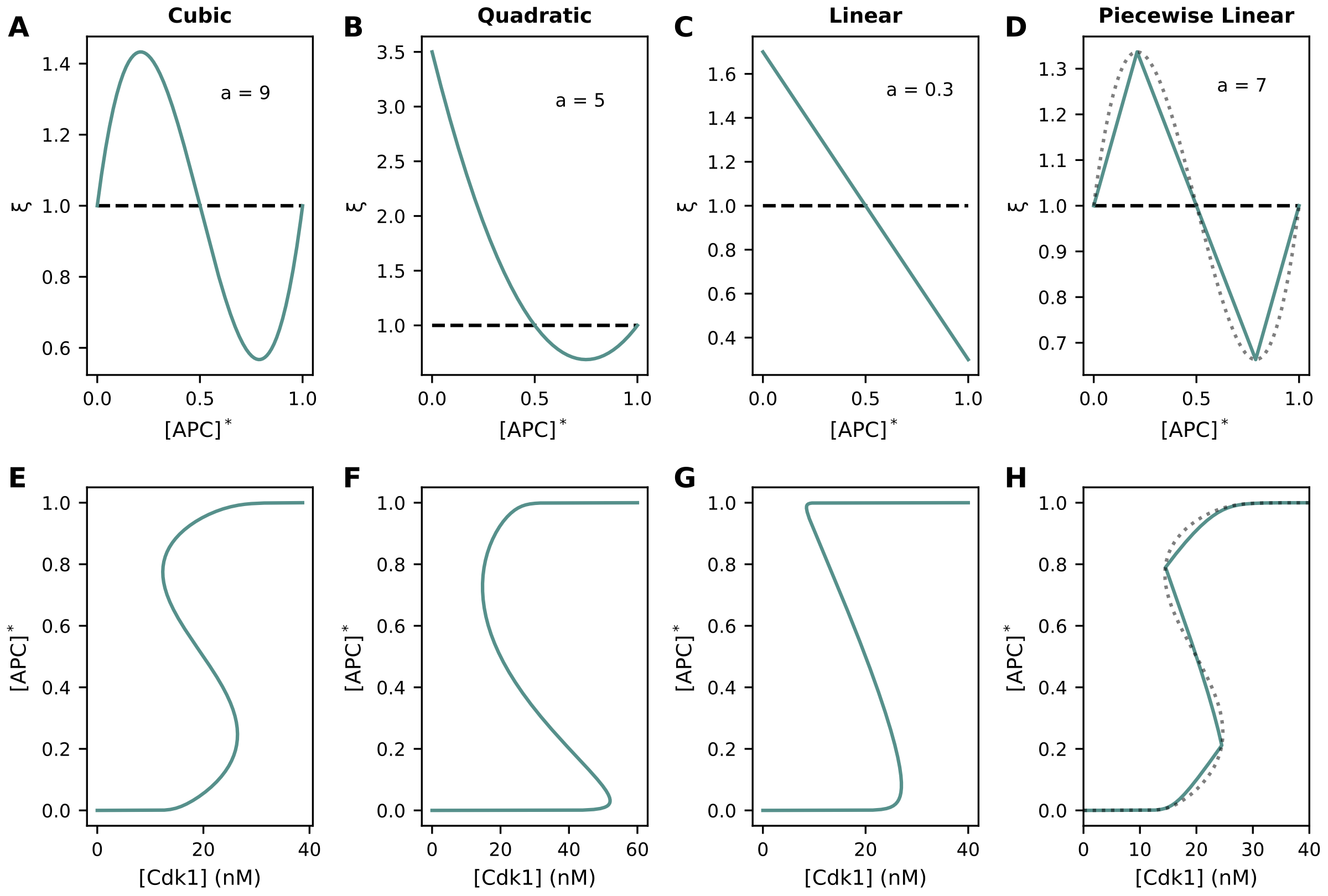

Supplement: S1 Fig — (A-D) Except from the cubic definition of ξ in the main text, alternative definitions can be used. An example of a quadratic function would be ξ([APC]*) = 1 + αapc([APC]* − 1)([APC]* − r). For a linear function, one possible definition would be ξ([APC]*)=1−αapcr−1([APC]*−1)+αapc. The equation for the piecewise linear approximation is given in the Methods section of the main text. In each case, r = 0.5. (E-H) Corresponding response curves obtained by multiplying a Hill function (K = 20, n = 15) with the scaling functions in panels A-D. (TIFF) [file pcbi.1009008.s002.tiff]

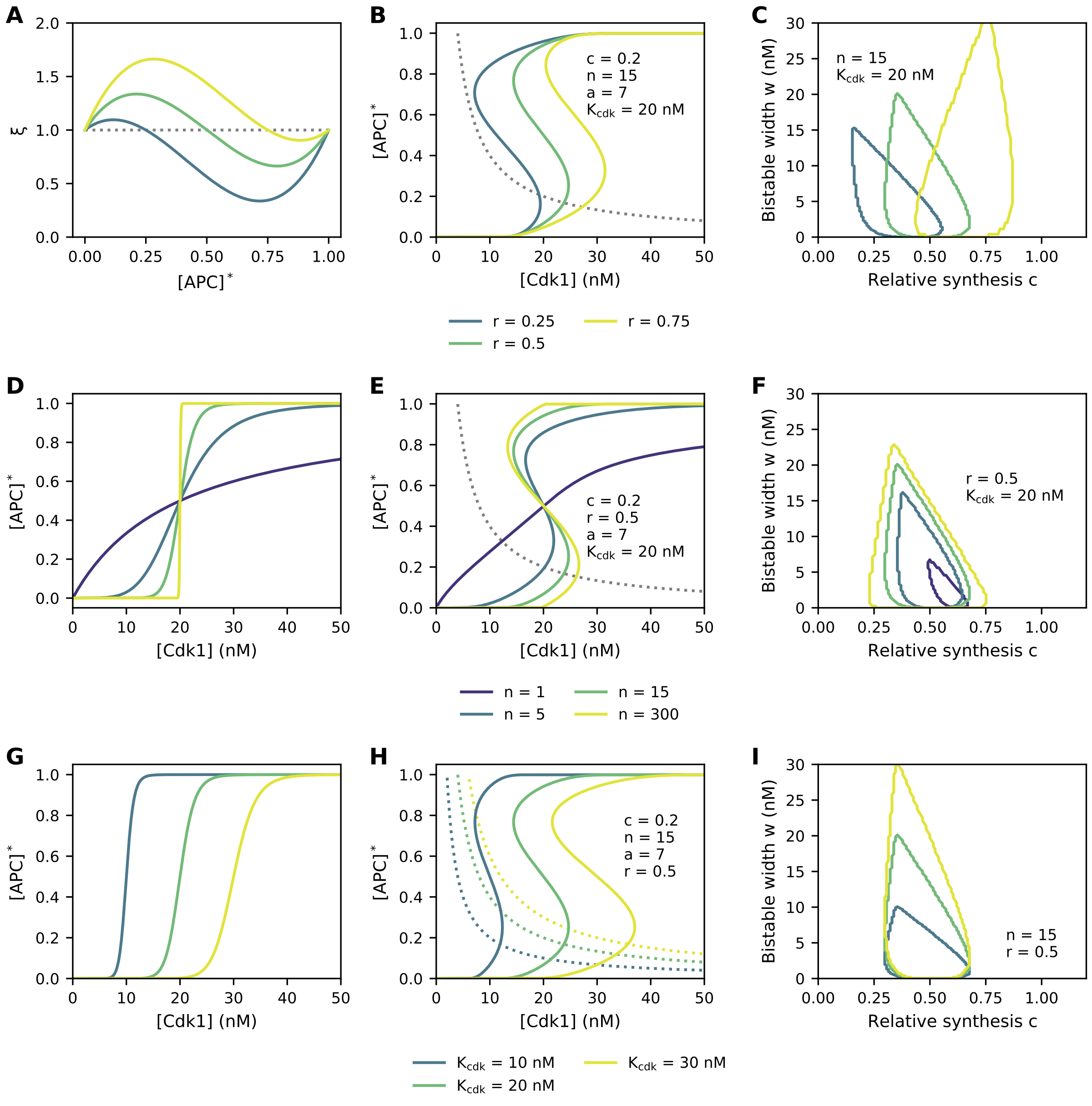

Supplement: S2 Fig — (A-C) Effect of parameter r on the scaling function ξ (A), S-shaped response curve in the phase plane (B) and oscillatory region in the parameter space (C). (D-F) Effect of the Hill coefficient n on the original ultrasensitive response (D), the derived S-shaped response curve in the phase plane (E) and oscillatory region in the parameter space (F). (G-I) Effect of threshold K on the original ultrasensitive response (G), S-shaped response curve in the phase plane (H) and oscillatory region in the parameter space (I). (TIFF) [file pcbi.1009008.s003.tiff]

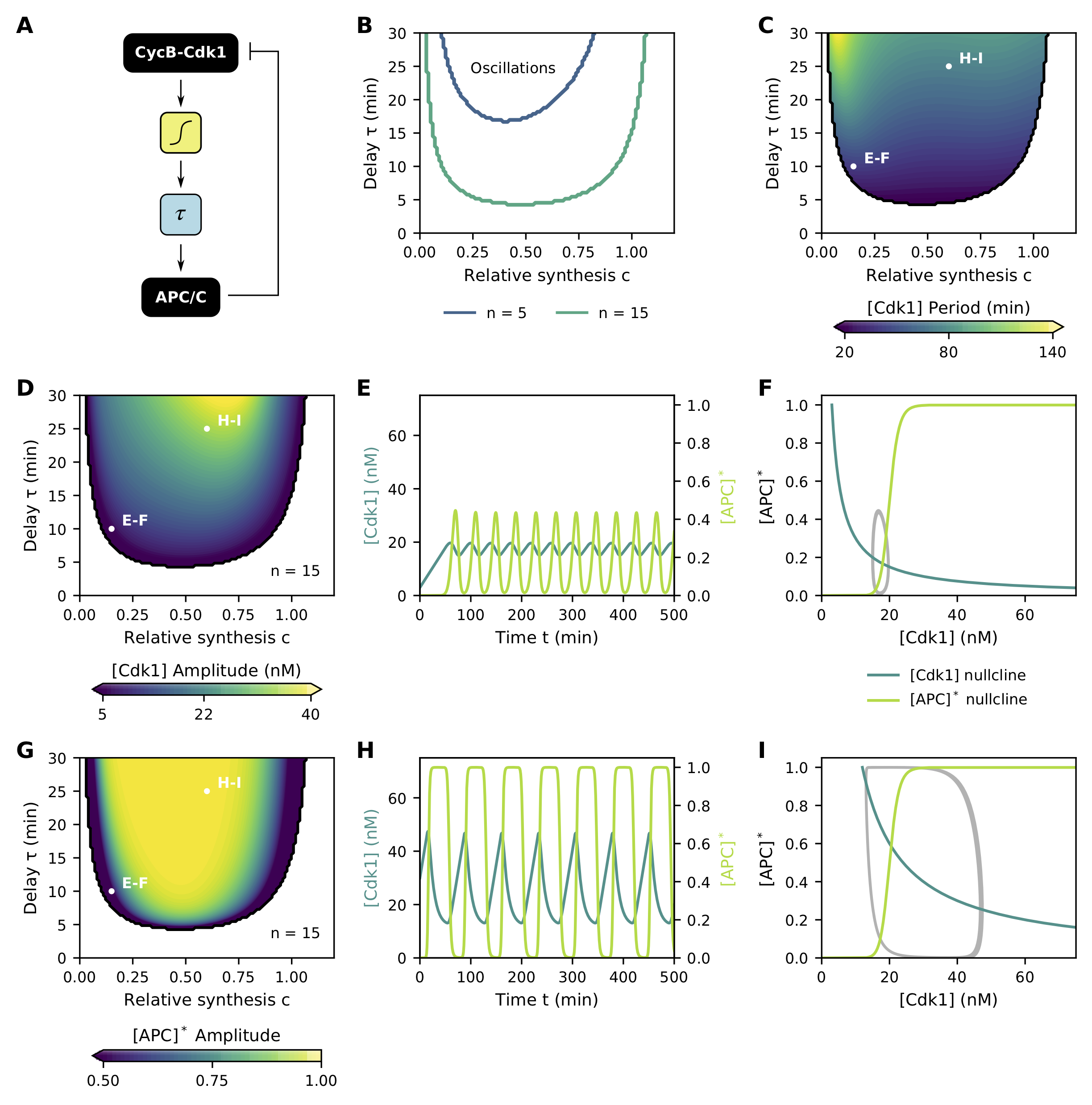

Supplement: S3 Fig — (A) Block diagram of the ultrasensitive, delayed negative feedback network. (B) Oscillatory regions for different values of the Hill coefficient n. (C) Period of the [Cdk1] oscillations as a function of the relative synthesis rate c and time delay. (D,G) [Cdk1] and [APC]* amplitudes as a function of the relative synthesis rate c and time delay. (E,F,H,I) Time traces and phase planes for indicated parameter values. (TIFF) [file pcbi.1009008.s004.tiff]

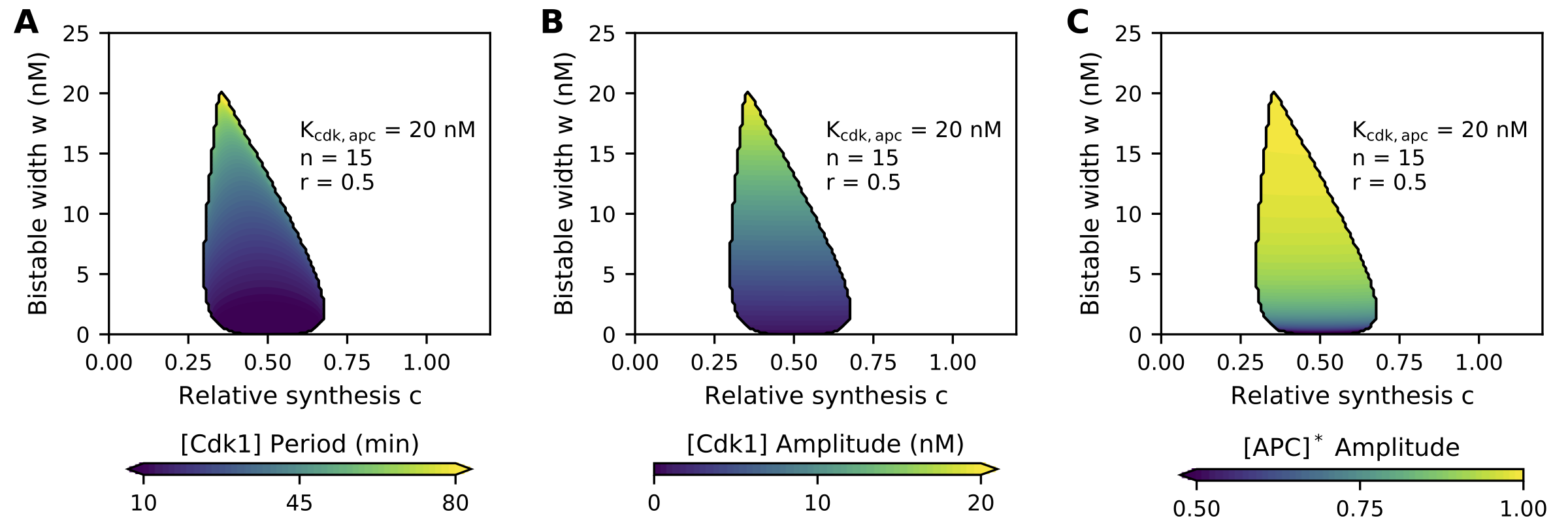

Supplement: S4 Fig — (A) [Cdk1] period as a function of the relative synthesis c and the width of the S-shaped region. (B) [Cdk1] amplitude as a function of the relative synthesis c and the width of the S-shaped region. (C) [APC]* amplitude as a function of the relative synthesis c and the width of the S-shaped region. (TIFF) [file pcbi.1009008.s005.tiff]

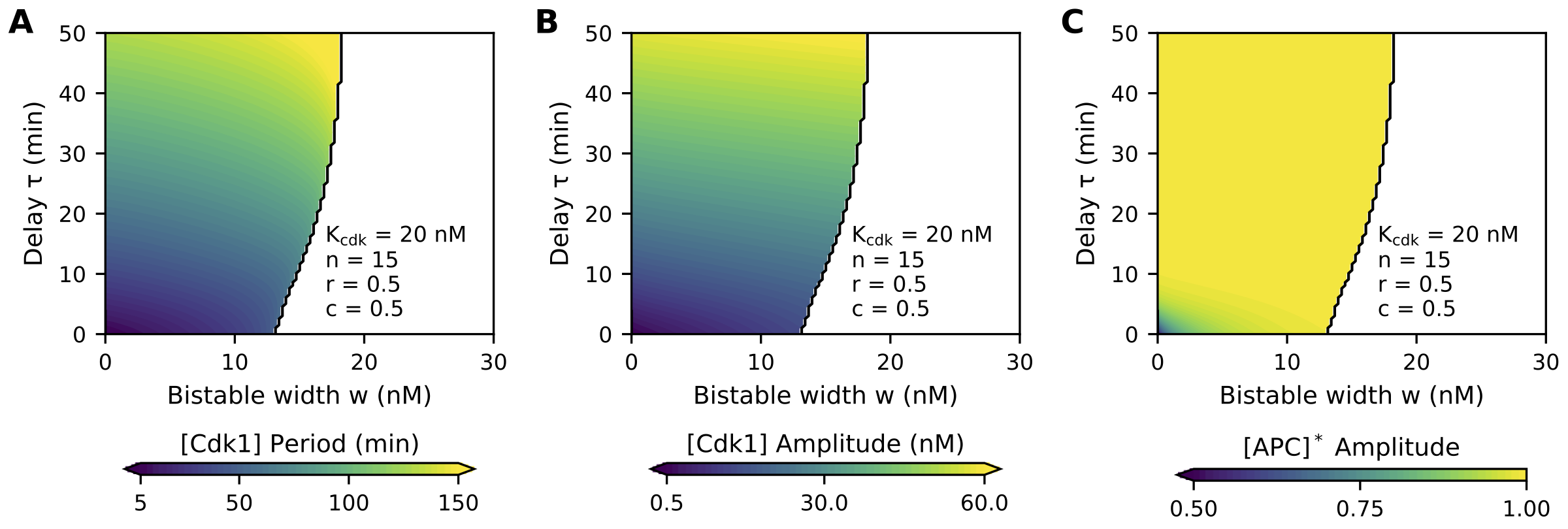

Supplement: S5 Fig — (A) [Cdk1] period as a function of the width of the S-shaped region and delay. (B) [Cdk1] amplitude as a function of the width of the S-shaped region and delay. (C) [APC]* amplitude as a function of the width of the S-shaped region and delay. (TIFF) [file pcbi.1009008.s006.tiff]

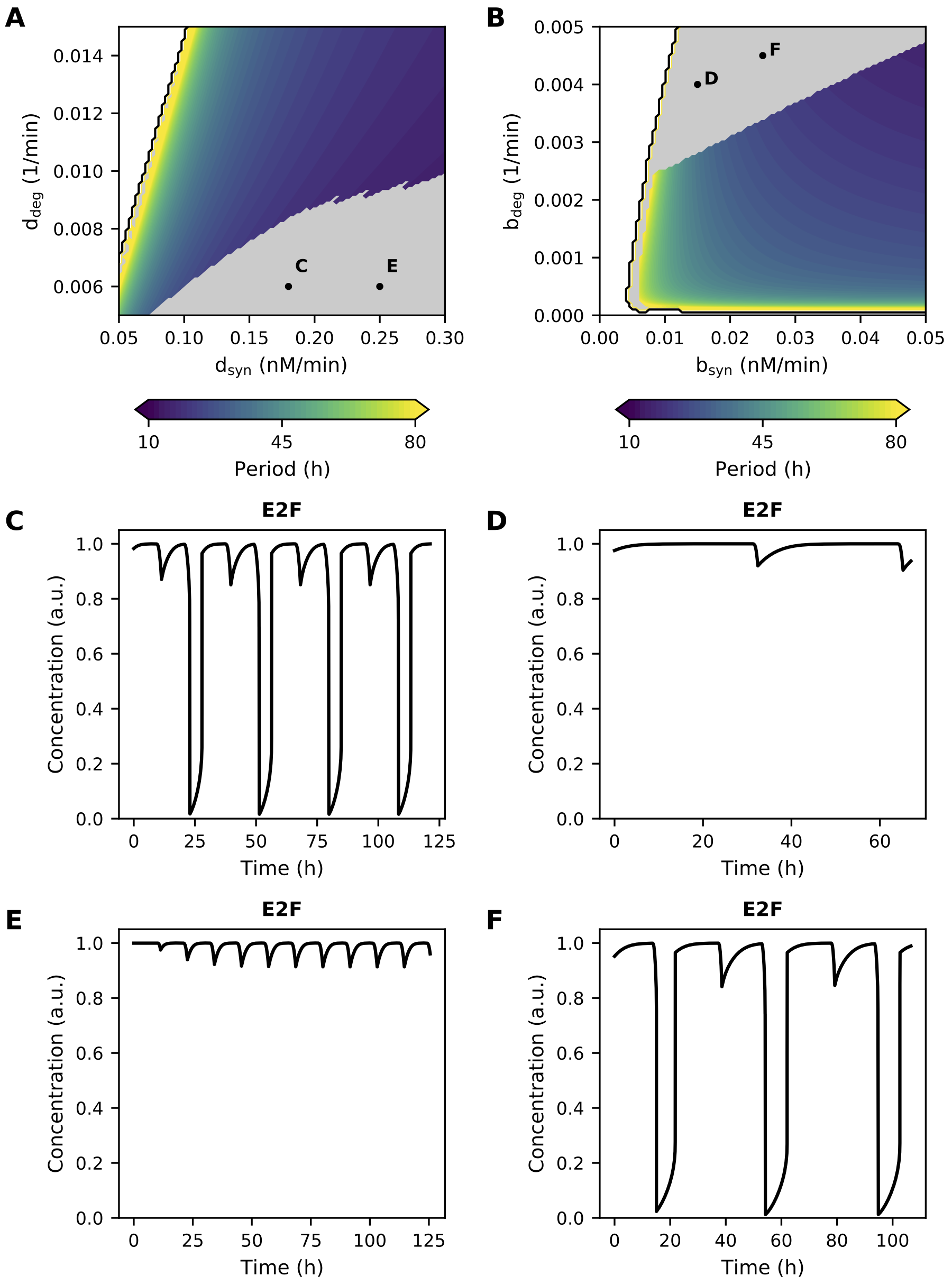

Supplement: S6 Fig — In Fig 7 in the main text we indicated grey regions in parameter space for which irregular oscillations were observed. Here, we show time traces of [E2F]* for such irregular patterns. (TIFF) [file pcbi.1009008.s007.tiff]

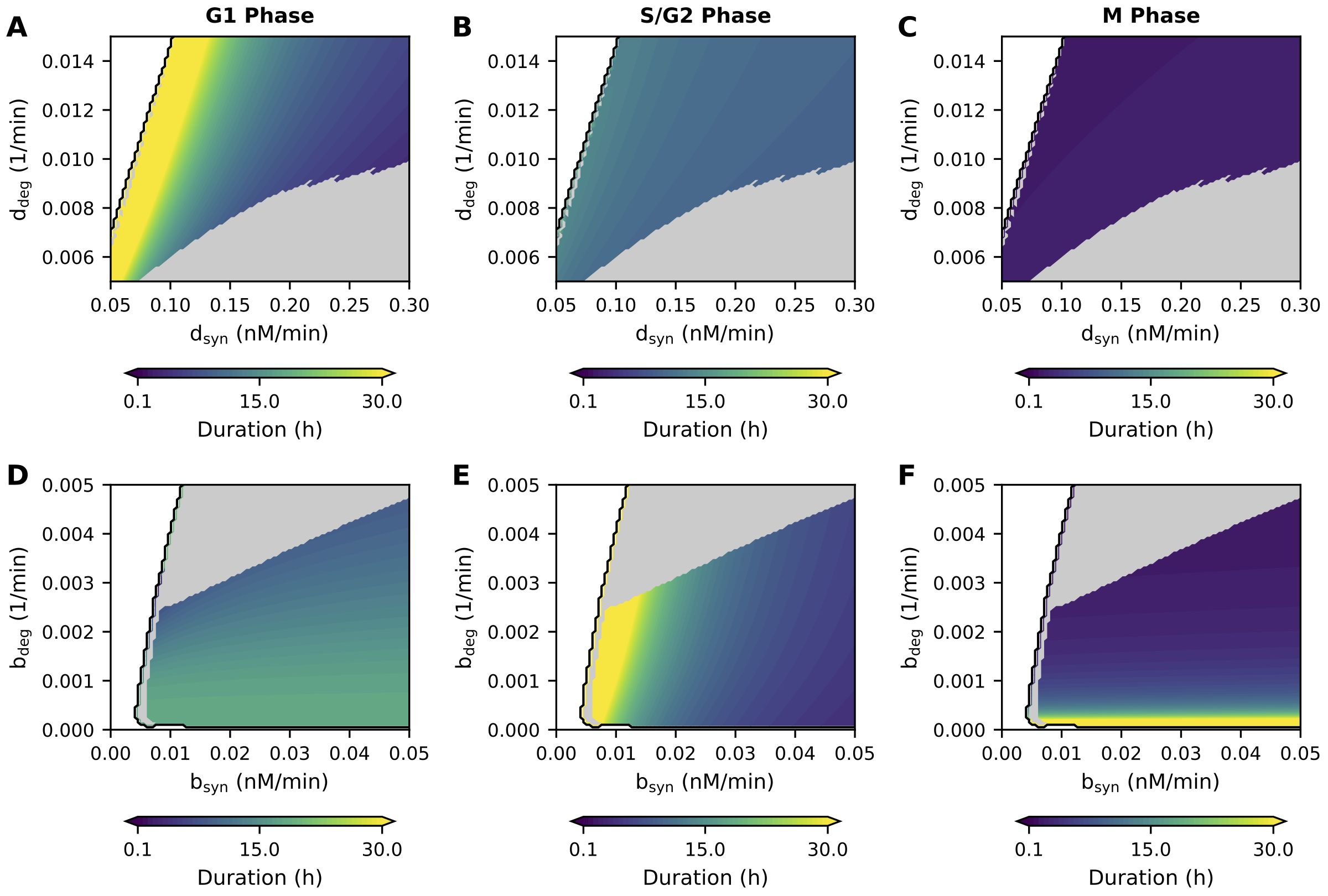

Supplement: S7 Fig — In Fig 7 in the main text we showed the effect of changing synthesis and degradation rates on the overall length of the cell cycle. Here, we separate the effects on the different cell cycle phases. White areas represent regions where no oscillations can be observed, while for the grey areas irregular oscillations exist. (TIFF) [file pcbi.1009008.s008.tiff]

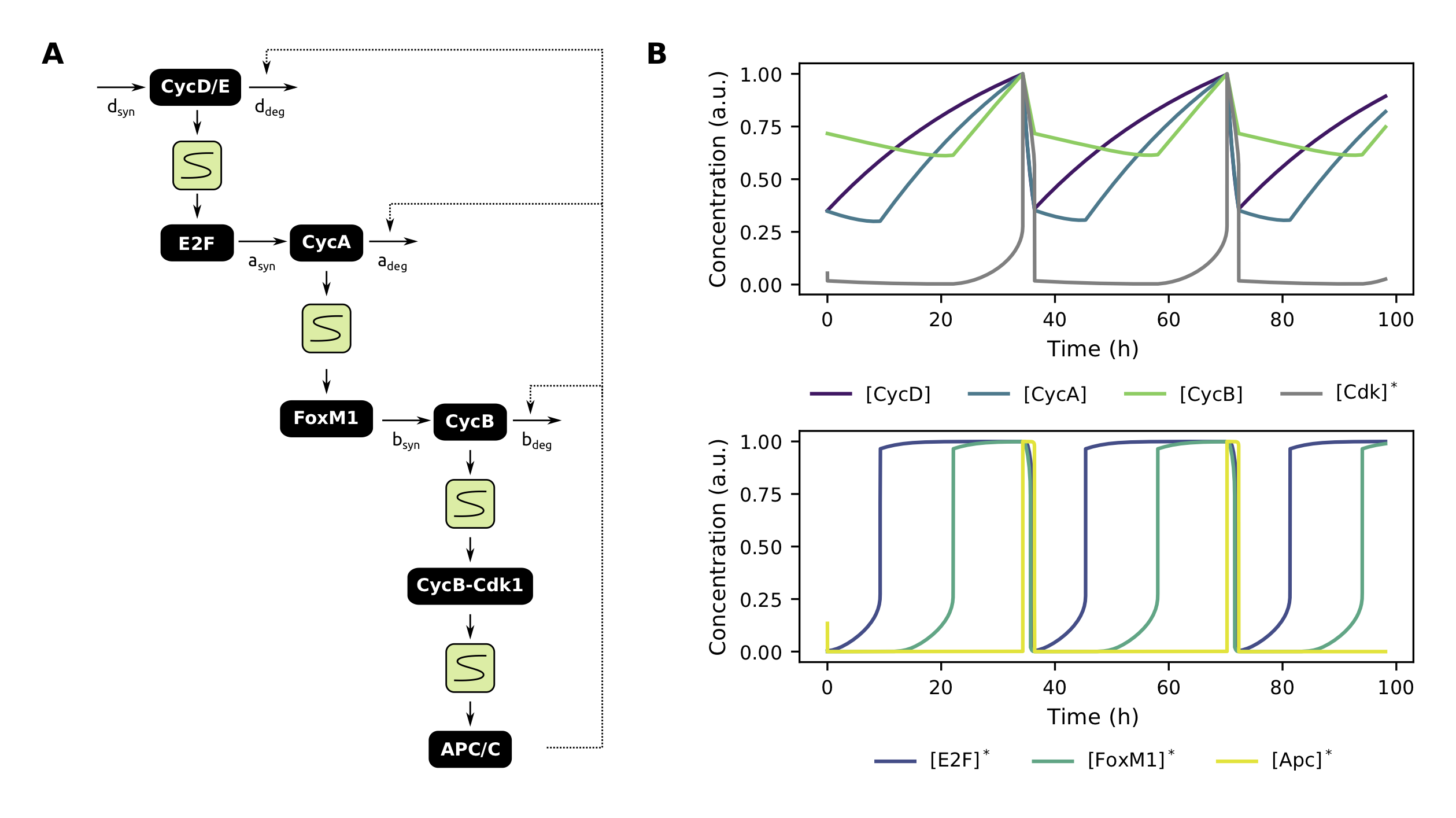

Supplement: S8 Fig — In Fig 7 in the main text we represented the cell cycle as a chain of three bistable switches. Here, we extended this model by including the hypothetical switch of FoxM1 activity with respect to CycA levels. (TIFF) [file pcbi.1009008.s009.tiff]

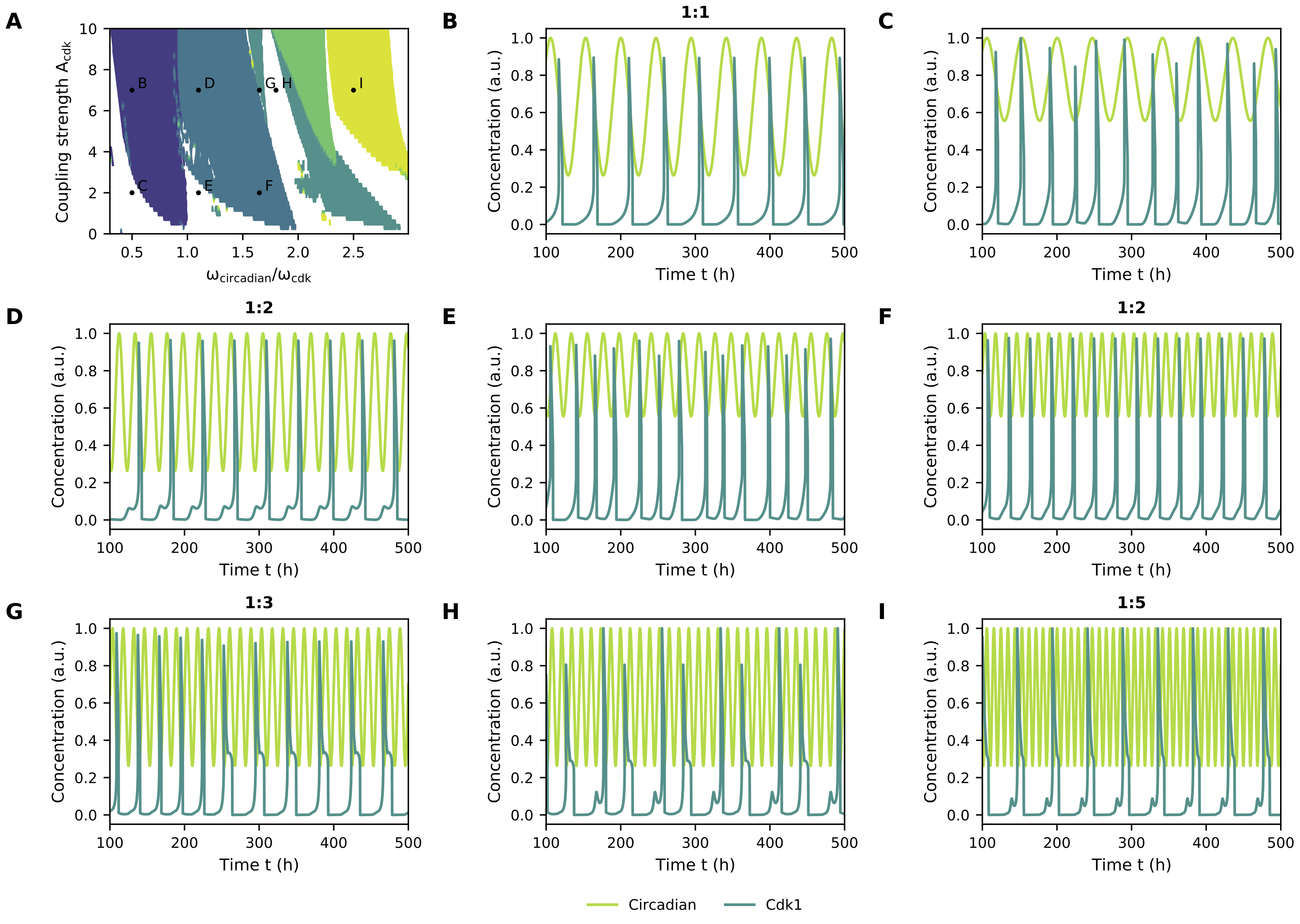

Supplement: S9 Fig — Time traces showing the absence or presence of p:q phase locking (with p and q in {1,2,3,4,5}) between the cell cycle and the circadian clock for several parameter combinations. Related to the Arnold tongues shown in Fig 8. (TIFF) [file pcbi.1009008.s010.tiff]
