# Supplementary material for: A modular approach for modeling the cell cycle based on functional response curves
Source: PLoS Comput Biol. 2021 Aug 11;17(8):e1009008. doi: 10.1371/journal.pcbi.1009008 (PMC8382204; doi:10.1371/journal.pcbi.1009008)
Supplement: Attachment — Submitted filename: Review of Gelens.pdf [file pcbi.1009008.s012.pdf]

Review of ‘Modular Approach for Modeling the Cell Cycle’

The manuscript by De Boeck, Rombouts & Gelens presents an intriguing new proposal for including bistable response curves in ‘phenomenological’ models of cellular regulatory networks, like the CDK control system in the eukaryotic cell cycle. The paper is clearly written, for the most part, well-illustrated and provides an expansive reference list. The editor(s) might take into account that this manuscript combines elements of an original research article (on turning sigmoidal response functions into S-shaped response functions) with a lengthy review article on mathematical modeling methods for molecular control systems in general and cell cycle control systems in particular. The combination is effective, in my opinion.

Comments and suggestions:

1. Mostly the authors speak of the ‘Hill function’, but occasionally of the ‘Hill equation’. It is a function, not an equation. Also, sometimes they refer to the ‘Hill coefficient’ when they mean the ‘Hill exponent’.

2. Lines 142-147. ‘a lag time…can be explicitly modeled by setting Out(t) = In(t-tau). This turns the equation (??) into a delay differential equation.’ These sentences are confusing. What the authors mean to say is that ODE (2) is converted into a DDE by replacing In(t) by In(t-tau).

3. Line 168. ‘eps_apc’ is a time-constant; ‘1/eps_apc’ is a rate constant.

4. The section ‘Asymmetric S-shaped APC/C nullclines…’ is a distraction, in my opinion. Why introduce a piecewise-linear approximation and set n=300? Seems weird to me. Why not just change the parameters r and alpha simultaneously to position the fold points as desired? I suggest removing this section or putting it in ‘Supplementary Material’.

5. The next section ‘S-shaped module reproduces mass-action model’ is also a distraction. Remove, or put it in ‘Supplementary Material’.

6. The section ‘Cell cycle as a chain of interlinked bistable switches’ is central to the manuscript, but I have some misgivings.

a. The model would be more useful if it included CycE and CycA. Not hard to do.

b. Although the model is set up as a sequence of bistable switches (falling dominoes), it functions as a limit cycle oscillator…not much different from the frog egg oscillator. In particular, there is no requirement for cell growth. This type of model (of which there are already too many in the literature) is severely limited in describing progression through the eukaryotic cycle. For example, line 408 ‘The duration of the different phases [and of the entire cell cycle] depends on the values of production and degradation rates.’ But this isn’t true. By mutations, one can easily change production and degradation rates of many components of the mechanism without changing the overall cell cycle time, which is determined by the growth conditions (nutrition, growth factors) of the cell culture. A mutation may lengthen the duration of S/G2/M, but this will be compensated by a shorter G1 phase to keep the total cell cycle time constant. All limit-cycle models of somatic cell cycles fail to account for this basic feature of the cell cycle. [This is a MAJOR criticism of the paper; it should be addressed by the authors.]

7. The next section, on cell cycle checkpoints, helps to clear up the function of the bistable switches. But…

a. The discussion of cell cycle (CC) control by circadian rhythms (CRs) leaves much to be desired. It is based on the assumption that the cell cycle, like CRs, is controlled by a limit cycle oscillator, which it isn't. If it were, then coupling of the two would lead to synchronization, but I don't know of any experimental studies that demonstrate that CC-CR coupling can induce changes in the natural frequency of either rhythm. On the contrary, there is a revealing study by Charvin, Cross & Siggia (PNAS, 2009) of the budding yeast cell cycle driven by periodic expression of G1 cyclins. The cell cycle does not synchronize to the driving rhythms but shows temporary phase-locking before it breaks away and relocks at a new phase. I suspect the same sort of behavior is displayed by cell cycles driven by CRs: intermittent phase-locking of mitosis to a particular phase of the CR.

b. Figure 9H is strange: CR of period 12 h, 19 h or 31 h??

c. Should include a reference to Yan & Goldbeter (J R Soc Interface, 2019).
