# Supplementary material for: A modular approach for modeling the cell cycle based on functional response curves
Source: PLoS Comput Biol. 2021 Aug 11;17(8):e1009008. doi: 10.1371/journal.pcbi.1009008 (PMC8382204; doi:10.1371/journal.pcbi.1009008)
Supplement: Attachment — Submitted filename: PCOM_Modular_SJ_edit - (2).pdf [file pcbi.1009008.s013.pdf]

The manscript “A modular approach for modeling the cell cycle based on functional response curves” proposes a way to reduce the complexity of the model by replacing the detailed reaction mechanisms of modules (ultrasensitive responses, S-shaped responses and time delays) in the model. The proposed simple approach to reduce the complexity of the model is interesting and useful to investigate complex systems. I believe that the manuscript fits well to the aim of PLOS Computational Biology.

Major comments

1. While the manuscript focuses on illustrating how to use the approach with various examples, the validity of this approach is rarely discussed. That is, for the validation of this approach, one expects the comparison between the dynamics of models based on mass-action kinetics having one of ultrasensitive responses, S-shaped responses and time delays and their simplified models with the proposed approach. Thus, please illustrate which parts of the dynamics of the original model are quantitatively or qualitatively captured by the simplified model. Importantly, please also illustrate which part of the dynamics of the original model cannot be captured by the simplified model. This will provide a guideline for the readers to use the proposed approach.

Deleted: either

Deleted: Then,

Deleted: is

Furthermore, from Fig. 3 to Fig. 8, various features of the systems with ultrasensitive responses, S-shaped responses and time delays are predicted with the simple models. Please describe that which predicted features match with previously known ones and which features are newly predicted. The matched features will indirectly support the validity of this approach and the new features will show the usefulness of the approach.

2. The proposed approach is based on Hill-functions. A recent review (Kim and Tyson, PLOS Com Biol (2020)) illustrates the risk of using such functions to describe protein-protein interactions. In particular, the key modules and examples discussed in this manuscript are usually generated by protein-protein interactions. Please discuss under which condition the proposed approach can be applicable to describe the modules generated by protein-protein interactions and whether the approach can be extended via the function based on tQSSA rather than Hill functions.

3. Authors argue that asymmetric bistable APC/C nullclines slow down APC/C (in)activation. However, the dynamics author used can also be slowed down if one uses symmetric APC/C nullclines with smaller $[\mathrm{APC}]^*_{RF}$ value and larger $[\mathrm{APC}]^*_{LF}$ value. Thus, asymmetry of nullclines seems not required to obtain slow dynamics.

Minor comments

1. In Figure 6, oscillations with large amplitudes are considered as robust oscillations. Please clearly state that for what type of perturbations, the oscillator is robust.

Deleted: a

Deleted: is

2. The legend of Fig 4B mis-illustrates figures.

3. In figure 6DE, relative synthesis rate c is not specified.

4. Not only for DNA damage and circadian clock coupling, but also for the circadian clock itself, the bistability plays the important role (Beesley S et al, PNAS (2020)).
