# Supplementary material for: A modular approach for modeling the cell cycle based on functional response curves
Source: PLoS Comput Biol. 2021 Aug 11;17(8):e1009008. doi: 10.1371/journal.pcbi.1009008 (PMC8382204; doi:10.1371/journal.pcbi.1009008)
Supplement: Attachment — Submitted filename: Letter_Referees.pdf [file pcbi.1009008.s014.pdf]

Reviewer 1 comments

In this work, a mathematical model of cell cycle regulation is developed by combining three functional modules (ultrasensitive, bistable and time delay). These modules are not derived from a detailed description of the underlying biochemical events, but provide a phenomenological description of a given desired behavior. It is shown that, by combining multiple switches and by including time delay, the proposed cell cycle model is able to describe events such as DNA damage and coupling to the circadian clock. In my opinion, the proposed strategy of combining multiple phenomenological modules to describe complex behaviors is promising and deserves investigation.

The major concerns about the reasoning and results are listed below, and concern the presentation and technical correctness of some derivations:

Our response: We thank the referee for carefully reading our manuscript and providing constructive feedback. We are pleased to see that our work is considered promising and deserving of investigation. Based on the feedback, we have improved the presentation of our results and we have addressed all technical concerns as well.

1. Abstract, Introduction (page 4, lines 113-117) and Results (page 5, line 122): even if the difference between an ultrasensitive and an S-shaped curve will be clarified later in the text, at this point, such a difference is, in my opinion, not clear. Indeed, an ultrasensitive response described by a sigmoidal function (e.g. the Hill function with Hill coefficient greater than 1) is itself an S-shaped curve.

Our response: We agree with reviewer 1 that the terminology related to bistable/S-shaped response curves can be confusing. Unfortunately, we are not aware of any other option to describe such a response, except from S-shaped or bistable. As previous reviewers commented on the fact that describing an S-shaped response as being bistable is not entirely correct (a statement we agree with), we opted for using S-shaped. Note though that in our opinion an ultrasensitive curve is not really S-shaped, as we consider that an S shape really requires "two bumps", which an ultrasensitive curve does not have.

2. Page 13 (system (iii)). As explicitly mentioned by the authors (see lines 334-335), the right hand side of the second ODE seems to not correspond to any of the modules (ultrasensitivity, S-shaped, delay) introduced in the first section, since the Hill-like equation is multiplied by [CycB]. Then, the model cannot be seen as a composition of the previously introduced modules. Furthermore, the choice of denoting the total concentration of CycB-Cdk1 complexes and the concentration of activated CycB-Cdk1 complexes by [CycB] and [Cdk1], respectively, (lines 330-331), is very confusing.

Our response: We apologize for this haphazard explanation of the variable names. The variable [CycB] equals the total amount of CycB levels, which however also represents the total concentration of CycB-Cdk1 complexes due to rapid binding. To avoid any confusion, we now refer to [CycB] as the total amount of CycB in the main text. The discussion of the equivalence with the total concentration of CycB-Cdk1 complexes is deferred to the Methods, where we use it to derive the equations for system (iii) and try to more clearly explain the reason for multiplying the RHS of the ODE by [CycB]. This latter multiplication indeed distinguishes system (iii) from the other systems in the text and might lead to some confusion. However, if one considers the steady-state response of an output being normalized to its total concentration, all systems in the text behave identical: their steady-state will be an S-shaped curve (as presented in Fig. 9). For system (i), the 'total concentration' is given by the constant value [APC_{tot}]. Therefore, the steady-state expression for [APC]/[APC_{tot}] can directly be introduced in the ODE as was done in Eq. 6. In system (iii), the 'total concentration' is itself a variable (i.e. [CycB], for reasons explained above) rather than a constant. Directly incorporating [Cdk1]/[CycB] into the ODE would therefore require the use of the quotient rule for differentiation, greatly complicating the appearance of the ODE. Alternatively, and arguably easier, one can transfer [CycB] to the RHS of the steady-state expression, prior to incorporating it into the ODE, as was done here. This, however, does not impose any technical problems as

multiplication by [CycB] just changes the shape of the S-shaped curve, but maintains the existence and width of the S-shape.

3. Pages 22-23. The ultrasensitive response [Cdk1]/[CycB] is not the same ultrasensitive curve as before (e.g., page 21, line 607 and below). The most remarkable difference is that, as noted by the authors (page 23, lines 632-633), with this expression, the Cdk1 curve does not saturate. On the contrary, it grows indefinitely. Then, its inverse is not exactly an S-shaped curve (I have generated a MATLAB plot to visualize this). Consequently, the plots of Figure 10 are not valid anymore and, indeed, in this case, the inverted ultrasensitive curve of Figure 10, panel (B) is not exactly an S-shaped curve. The reason for this behavior is mentioned by the authors at lines 628-629: "[CycB] is not a constant and therefore, the rate of change of [Cdk1]/[CycB] would need to be given by the quotient rule for differentiation."

Our response: The steady-state response of [Cdk1] with respect to [CycB] indeed differs from the ultrasensitive response shown in Fig. 9A, as the curve does not saturate. The steady-state response of [Cdk1]/[CycB] on the other hand, does saturate at y=1 owing to the normalization for the total concentration (see also comment 2). We now emphasized in the caption that Fig. 9 only applies to normalized responses and refer to the figure more consistently in our explanation in the text.

4. Page 24. It is clear how the expression of the non-dimensionalized inverted bistable response curve x has been computed. However, both the independent variable y and the dependent variable x are functions of [CycB], more precisely, y is a function of x. How is it possible to compute the derivative of x with respect to y given the fact that y a function of x?

Our response: We thank reviewer 1 for pointing out this lack of explanation and added the reasoning behind this approach in supplemental information. In case x = [CycB] and y = [Cdk1]/[CycB], we are actually interested in the width of the [Cdk1] response, not the one of [Cdk1]/[CycB]. Letting z = [Cdk1], we thus need to solve dx/dz = 0. As x is a function of y, and y a function of z, we use the chain rule of differentiation to find dx/dz = (dx/dy)(dy/dz). From this, it is seen that dx/dz becomes zero whenever dx/dy is zero. Therefore, finding the roots of dx/dy (as was done in the text), allows us to find the roots of dx/dz.

5. Page 26, lines 671-672. "Default parameter values were manually screened so that oscillations with biologically relevant periods were obtained (Table 2)." It seems that the cyclins thresholds are the parameters that have been manually screened to generate oscillations. How are the thresholds of cyclins related to one another with respect to the biologically relevant periods? Are these thresholds compatible with the experimentally measured values?

Our response: The chosen thresholds are indeed in line with experimentally measured levels (refs 11, 15) or in line with other models (refs 19, 56).

6. Page 26, lines 680-682. The authors state that "This number was chosen based on experimental measurements that demonstrated how growth factor stimulation increases CycD levels between 4 and 20 fold [98, 99]." Could the authors be more precise with respect to the number used and to what it means in the context of testing the effect of the restriction point?

Our response: In the Methods, we added the reason for choosing a 10-fold reduction (intermediate value between 4 and 20) and mention that also other values would work.

In addition, the following aspects require substantial explanations:

7. Page 4, lines 82-106. The difference between the two strategies is now clear: the first strategy consists in omitting some of the reactions, while the second strategy simplifies the model by introducing some assumptions (e.g. the steady state assumption). Can the use of phenomenological expressions be considered as a third strategy?

Our response: In a way, the phenomenological expressions are like the second strategy as they lump together several underlying molecular reactions and are based on the principle of time scale separation. However, the phenomenological approach differs in the way the expressions are derived: whereas the second strategy starts from mass action models and introduces certain assumptions, the phenomenological expressions are directly taken from either experimental data or from the output of full mass-action models. These similarities and differences are now shortly discussed at the end of section ‘The S-shaped module reproduces the behavior of mass-action models’.

8. Page 11, (system (ii)). The role of delays τ_1 and τ_2 in the ODEs of (system (ii)) is not clear. Whilst their meaning is clearly explained in lines 295-300, connecting these explanations to the way in which τ_1and τ_2 appear in the ODEs is not straightforward. In addition, when τ_1 and τ_2 assume different values, the time delay in both ODEs becomes time-varying (since τ is a function of [APC]*); is this biologically reasonable?

Our response: We tried to clarify these points by reorganizing this section. Expressing the delay as a function of [APC] to arrive at state dependent delays is now introduced in the text before the equations are given. Also, the effect of the different terms in the expression of the state dependent delay is better explained, by explicitly mentioning how the used Hill function becomes zero or one for [APC] < 0.5 and [APC] > 0.5, respectively. As a possible biological situation in which both time delays may be different, we mention how the activation of APC/C may take longer than its inactivation (for instance due to the multistep nature of this activation, similar as in ref 16).

9. Page 14, Figure 7. Comprehension and interpretation of the plots seems to be rather hard. Moreover, panel (B) refers to both system (iii-b) and (iii-c): does this mean that the two systems exhibit equal [Cdk1] amplitude?

Our response: Both systems correspond to different parts of the plot. The horizontal section at $W_{cdk,apc} = 0$ (i.e. bistable width of 0, hence ultrasensitive response between CycB-Cdk1 and APC) corresponds to system (iii-b), while the parts of the plot for which $W_{cdk,apc} > 0$ correspond to system (iii-c). This is now explicitly stated in both the text and caption. Furthermore, we agree that the plots were rather hard to interpret. For that reason, we have changed the figure entirely in order to make it more comprehensible, while not changing the main messages of the figure, which are that oscillations are more robust with two underlying switches and that there exist two modes of oscillation (high amplitude vs low amplitude in Cdk1).

10. Page 15. Several concerns arise regarding systems (iii-a), (iii-b) and (iii-c). First of all, at line 340, it should be clarified that the label “system (iii-a)” refers to Figure 7A. At lines 341-344, the parameter d has been introduced; it seems to be confusing to define the relative synthesis first as c and then as the ratio between c and d? Also, the considerations at lines 351-357 are not clear.

Our response: The reference to Fig 7A is now included when first mentioning system (iii-a). We see how the usage of both c and c/d may be confusing. The relative synthesis is always defined as the positive cyclin production term in the non-dimensionalized ODE, and was defined as c in system (i), but naturally becomes c/d in system (iii) (see Methods). As such, the blue shaded area can directly be compared with the green contour line in Fig 6A (by just plotting c in Fig 6A, the blue shaded area would be twice as wide, giving a distorted picture of the effect of the synthesis rate). We tried to clarify the meaning of the ‘relative synthesis’ in the text and Methods and removed the labels c and c/d from the plots in Fig 6 to avoid confusion.

11. Pages 17 and 19 ODEs are missing for the model in which the circadian regulation of Wee1 has been introduced (see lines 460-461). Also, the concept of Arnold tongues (lines 473-490) would require additional explanations. Finally, the expression “the strength of the coupling (here Acdk, i.e. the extent to which the [Cdk1] activation threshold is shifted to higher [CycB] levels over one period)” (line 471-472).

Our response: The ODEs of the circadian regulation are the same as the ones for Fig. 7 and the other panels in Fig. 8. We now specifically refer to the Methods for both the ODEs and the details on how we periodically altered

the threshold of the S-shaped response (which was done by modeling the parameter α as a sine wave). The coupling strength is now explicitly referred to as 'the amplitude of the sine wave used to model α' in both the text and figure caption. Regarding the concept of Arnold tongues, we now first conceptually discuss how phase-locking can be visualized before mentioning 'Arnold tongues'. An in-depth discussion of Arnold tongues is beyond the scope of this paper, but we now included a reference for additional information regarding this topic.

12. Page 19, line 493. "Under the premise that the functionalities of these individual modules do not change when combined with each other". Unfortunately, this assumption is not always verified, as explained few lines below (see e.g. 499-500).

Our response: Indeed, as we explain in the text and as noted by reviewer 1, certain systems cannot be described by simply combining individual switches. In such cases, the approach discussed here cannot be expected to accurately represent the behavior of the actual system. In the first paragraph of the discussion, we now added some references that show how bistability itself allows for a modular approach, as well as a reference exploring the effect of coupling ultrasensitive modules.

13. Page 26. in the system equations for interlinked switches, what is the meaning of parameters δ_d and δ_b? It would be better to introduce the meaning of these parameters within the text, in addition to Table 2. These terms were present neither in (systems (ii)) nor in (system (iii)). Why were they added in the interlinked switches system? Furthermore, the right hand side of the fourth ODE does not correspond to any of the modules (ultrasensitivity, bistability, delay) introduced in the first section since, again, the Hill-like equation is multiplied by [CycB] (see the previous comment regarding (system (iii))).

Our response: We thank reviewer 1 for pointing this out. The δ parameters determine the basal degradation rate of cyclins independent of [APC] and were included here as some of the regulatory aspects controlling the cell cycle (specifically DNA damage in G1) act upon these parameters. This reasoning is now added to the text in the Methods section. Regarding the multiplication by [CycB], we refer to comment 2.

14 Page 6, lines 136-138. How have the curves in Figure 2B been obtained? I guess the authors have computed the inverse of the function in Eq. (1) (namely, Input as a function of Output) and then they have plotted this latter inverse function for different values of α. I would suggest to explain this explicitly.

Our response: We added the explanation and a reference to Eq. 1 in the caption of the figure.

15 Page 10, section "The S-shaped module reproduces the behavior of mass-action models": In this section, the authors claim (lines 269-270 and lines 289-290) to show that the modified Hill function (namely, the "phenomenological" expression in Eq (2), which provides an S-shaped response) and its piecewise linear approximation are able to reproduce the oscillatory behavior of the mass-action model for the PP2A-ENSA-GWL network. Is there any reason why the S-shaped response should be preferred to the mass-action model?

Our response: The use of the S-shaped response greatly simplifies the analysis of the system, especially when experimental values for the mass-action parameters are not available. For the PP2A-ENSA-GWL network for example, many of the rate constants are not known, and even if some parameter values have been experimentally measured, the estimated values can be quite different (see for example the concentration values in comment 16). In generating the model, the parameters thus needed to be screened manually to obtain relevant oscillatory patterns (also discussed in the Methods). For the mass-action model, all 11 parameters plus synthesis and degradation needed to be adapted to find such oscillations. For the S-shaped module however, less than half of this number of parameters needed to be screened. In fact, if one can fit the S-shaped response to an existing model or – arguably better – to an experimentally determined response curve (which can be easier to obtain than rate constants) and synthesis and degradation constants are fixed (again for example, in line with a previous model or data), only the time constant ε of the phenomenological model needs to be manually screened. The choice of the model also depends on the question under investigation. In answering the question 'How does

the width of the S-shaped response affects the oscillations?', an explicit parameter determining this width (as in the phenomenological approach) is likely preferable. If on the other hand, one is interested in answering 'What is the effect of changing a certain rate constant?' (for example, in line with an experimental study using an inhibitor or knock-out), a mass-action model seems the logical choice. These reasons are also discussed throughout the text and in the discussion.

16 Page 25, Table 1. The value of ENSA_tot used by the authors is equal to 40nM, while in reference [61] this value is equal to 1000nM. As the authors refer to [61], which is then the specific reason to change the value of ENSA_tot compared to that paper?

Our response: We thank reviewer 1 for pointing out this large discrepancy with other reports and no longer use the low $ENSA_{tot}$ concentration of 40 nM for the mass action model. However, the 1000 nM in reference [61] is at the high end of estimates found in literature. Others have reported ENSA concentrations of 500-1000 nM [62] and 150-300 nM [Mochida, 2013, Febs]. In line with these lower estimates, we now use a concentration of 200 nM for $ENSA_{tot}$. Accordingly, also the other parameters were changed. The amount of $PP2A_{tot}$ is 5-fold less than $ENSA_{tot}$ [62] and we assumed GWL_{tot} concentrations similar as $PP2A_{tot}$. Rate constants were again screened manually to find oscillations. These choices with their references are now explicitly mentioned in the text.

Finally, the following minor comments require rephrasing, adjustments and/or a better explanation of concepts:

17 Page 7, lines 166-167. "the rate of change of [APC]* is proportional to the difference between the current [APC]* level and its experimentally determined ultrasensitive steady state response". Does this mean that the term - [APC]* is not representing degradation?

Our response: Indeed, the ODE is derived by explicitly defining its steady-state response curve. Therefore, positive and negative terms do not necessarily correspond to biological production and degradation terms. We now explicitly mention this when introducing system (i-a): 'Note thus how positive and negative terms in this ODE are not to be interpreted as actual production and degradation terms respectively'.

18 Page 9, line 235.What does "extrema of ξ([APC]^*)" mean?

Our response: This refers to the local extrema of the function. This section was omitted from the main text, but we now mention `local extrema' when we refer to the extrema of the function elsewhere in the text.

19 Page 11, lines 308-309. "Without a time delay, i.e. τ=0, system (ii) is identical to system (i)". This claim is trivial.

Our response: We rephrased this sentence to avoid the redundancy: 'Without a time delay, oscillations can be observed for intermediate values of the relative synthesis…'

20 Page 15, line 366. "and" should be replaced with "as".

Our response: done

21 Page 16: the concept of "oscillations orbiting around the response curves/bistable switches" is not clear; please, see the caption of Figure 8 ("Grey regions represent oscillations that are not orbiting around all three bistable switches.") and also lines 402-403 and lines 405-407

Our response: We now generically describe this type of oscillations as `irregular', without referring to the `orbiting around the bistable switches'. This concept of `orbiting around the bistable switches' however, is still used elsewhere in the paper, but is explicitly introduced when used the first time in relation to system (i-b).

22 Page 23, line 635. What does "apparent" mean?

Our response: `apparent' was added to stress the fact that the actual degradation is a second-order reaction, which is here modeled as a first order reaction. This in line with the classical definition of `apparent rate constants' and with the experimental measurements determining the value of b_{deg} (which are measured in units 1/min rather than 1/(M min)). To avoid any confusion, however, we removed it.

23 Page 27, line 696. Can the authors please indicate what do they mean with "shifted"?

Our response: As indicated, 'time shift' has to be interpreted as is the case when calculating an autocorrelation.

24 Supplemental Information, page 1, few lines below equation (1). "we will show that the steady state is linearly stable". Even if stability of the steady state is assessed through linearization, the expression "linearly stable" seems to be not correct, since model (1) is nonlinear.

Our response: We omitted 'linearly' and refer to a 'stable' steady-state as found by linearization of the system.

25 Page 4, line 108: please, replace "cycles" with "cycle" (singular).

Our response: done

26 Please replace "a S-shaped response/module" with "an S-shaped response/module": page 6, line 131; page 8, line 184 and line 186; page 11, line 308; page 15, line 385 and line 387; page 21, line 603; page 22, line 614;

Our response: done

27 Page 7, caption of Figure 3(E): please, replace "bistable [APC]* nullclines" with "S-shaped [APC]* nullcines".

Our response: done

28 Page 12, line 326: "a S-shaped response". Maybe, "bistable" would be more appropriate in this case.

Our response: done

29 Page 13, Figure 6(B): please, replace "bistable width" with "width of the S-shaped region".

Our response: done

30 Page 15, line 348: please, replace "bistable" with "S-shaped".

Our response: done

31 Page 15, lines 385-386: "can be depicted as a S-shaped module". I would suggest to rephrase as "can be depicted as an S-shaped (in this case, also bistable) module".

Our response: To avoid any confusion, we did not include this additional information.

32 Page 12, equation (12): in the third and the fourth differential equation, the parameter k_ass is present, whose value is not indicated in Table 1. Also, in the fifth differential equation, the parameter k_da appears, whose value is not present in Table 1.

Our response: Thank you for making us aware of these missing values, which are now added to the table.

Reviewer 2 comments

Review of 'Modular Approach for Modeling the Cell Cycle'

The manuscript by De Boeck, Rombouts & Gelens presents an intriguing new proposal for including bistable response curves in 'phenomenological' models of cellular regulatory networks, like the CDK control system in the eukaryotic cell cycle. The paper is clearly written, for the most part, well-illustrated and provides an expansive reference list. The editor(s) might take into account that this manuscript combines elements of an original research article (on turning sigmoidal response functions into S-shaped response functions) with a lengthy review article on mathematical modeling methods for molecular control systems in general and cell cycle control systems in particular. The combination is effective, in my opinion.

Our response: We thank reviewer 2 for considering our work to be intriguing, clearly written and well-illustrated (mostly), and effective in the combination of new research with reviewing the literature. The referee has raised interesting questions and suggestions, which we have addressed here below (and in the revised manuscript).

Comments and suggestions:

1. Mostly the authors speak of the 'Hill function', but occasionally of the 'Hill equation'. It is a function, not an equation. Also, sometimes they refer to the 'Hill coefficient' when they mean the 'Hill exponent'.

Our response: Thank you for highlighting this inconsistency in nomenclature. We now changed everything to 'Hill function' and 'Hill exponent'.

2. Lines 142-147. 'a lag time…can be explicitly modeled by setting Out(t) = In(t-tau). This turns the equation (??) into a delay differential equation.' These sentences are confusing. What the authors mean to say is that ODE (2) is converted into a DDE by replacing In(t) by In(t-tau).

Our response: We changed this explanation as suggested: "*For the last module, i.e. delay, a lag time τ between input and output of the system can be introduced. Replacing In(t) by In(t - τ) then converts Eq. 2 into a delay differential equation*."

3. Line 168. 'eps_apc' is a time-constant; '1/eps_apc' is a rate constant.

Our response: We now refer to '1/eps' as the rate constant of the equation: *"…which it approaches with a rate constant 1/ε".*

4. The section 'Asymmetric S-shaped APC/C nullclines…' is a distraction, in my opinion. Why introduce a piecewise-linear approximation and set n=300? Seems weird to me. Why not just change the parameters r and alpha simultaneously to position the fold points as desired? I suggest removing this section or putting it in 'Supplementary Material'.

Our response: We agree that this section is not crucial and can be distracting. Therefore, as suggested, we have omitted it from the main text.

5. The next section 'S-shaped module reproduces mass-action model' is also a distraction. Remove, or put it in 'Supplementary Material'.

Our response: We understand the viewpoint of the reviewer and we largely agree. We did not originally include this section in the text, but only added it after comments of several other reviewers (in an earlier submission). Therefore, it seems that this section can be important, depending on the specialization and background of the reader. Furthermore, we now also used this section to explain some limitations of our proposed approach.

6. The section 'Cell cycle as a chain of interlinked bistable switches' is central to the manuscript, but I have some misgivings.

a. The model would be more useful if it included CycE and CycA. Not hard to do.

Our response: In the main text, we focus on those bistable switches that have been experimentally measured, hence explaining why CycE and CycA were not originally included in the work. However, we follow reviewer 2 in his/her argument and see how incorporating additional switches might broaden the scope of the model. Furthermore, and as correctly noticed by reviewer 2, adding additional switches is not hard to do; in fact, the ease at which it can be done is one of the advantages of our modular approach. Therefore, we now incorporated a CycA-FoxM1 switch, of which the existence has been proposed (although not experimentally validated) elsewhere [Fu et al., 2008, Nat. Cell Biol.] and we emphasize how CycD and CycE work together in governing bistability in E2F activity. The figure below (which can now be found in supplemental information) shows this extension.

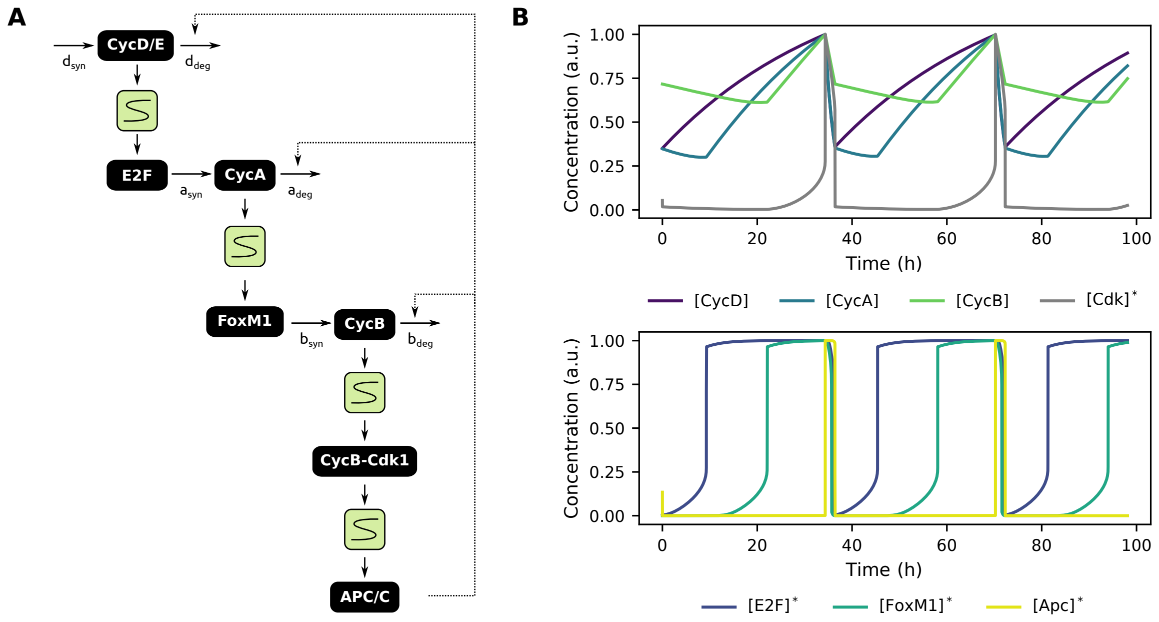

b. Although the model is set up as a sequence of bistable switches (falling dominoes), it functions as a limit cycle oscillator…not much different from the frog egg oscillator. In particular, there is no requirement for cell growth. This type of model (of which there are already too many in the literature) is severely limited in describing progression through the eukaryotic cycle. For example, line 408 'The duration of the different phases [and of the entire cell cycle] depends on the values of production and degradation rates.' But this isn't true. By mutations, one can easily change production and degradation rates of many components of the mechanism without changing the overall cell cycle time, which is determined by the growth conditions (nutrition, growth factors) of the cell culture. A mutation may lengthen the duration of S/G2/M, but this will be compensated by a shorter G1 phase to keep the total cell cycle time constant. All limit-cycle models of somatic cell cycles fail to account for this basic feature of the cell cycle. [This is a MAJOR criticism of the paper; it should be addressed by the authors.]

Our response: We thank reviewer 2 for pointing out this discrepancy between the proposed model and some experimental studies. Indeed, in Drosophila for instance, compensation in the duration of different cell cycle phases has been elegantly demonstrated [Reis et al., 2004, Cell] and especially in yeast, such compensatory mechanisms have been linked to size homeostasis (see for example [Garmendia-Torres et al., 2018, eLife]). In contrast, however, other studies revealed that cell cycle phases in mammalian cells are uncoupled, without the existence of any compensation [Chao et al., 2019, Mol Syst Biol]. In this latter study, one experiment showed that

increasing CycD synthesis entails a shortening of G1 phase and the overall cell cycle. This finding is in agreement with the results shown in Figure 7E. In reality, cells likely use both 'modes of action' in a context-dependent manner. Indeed, some studies demonstrated how during development, cells undergo a transition from 'compensating cell cycle phase duration' to 'not compensating' [Ogura and Sasakura, 2016, Developmental Cell]. Taken together, we agree with reviewer 2 that cyclin synthesis and degradation are not the only, and probably also not the most important, determinants of cell cycle duration. Accordingly, we elaborated a bit further on this in the text and included a figure (Fig 8A) showing how changes in cyclin synthesis can be compensated by other mechanisms to keep total cell cycle duration constant. To stay in line with the phenomenological approach used in this work, we modelled such compensatory mechanisms (e.g. the influence of cell size or extracellular nutrients) by changing the bistable switches, rather than incorporating them explicitly.

7. The next section, on cell cycle checkpoints, helps to clear up the function of the bistable switches. But…

a. The discussion of cell cycle (CC) control by circadian rhythms (CRs) leaves much to be desired. It is based on the assumption that the cell cycle, like CRs, is controlled by a limit cycle oscillator, which it isn't. If it were, then coupling of the two would lead to synchronization, but I don't know of any experimental studies that demonstrate that CC-CR coupling can induce changes in the natural frequency of either rhythm. On the contrary, there is a revealing study by Charvin, Cross & Siggia (PNAS, 2009) of the budding yeast cell cycle driven by periodic expression of G1 cyclins. The cell cycle does not synchronize to the driving rhythms but shows temporary phase-locking before it breaks away and relocks at a new phase. I suspect the same sort of behavior is displayed by cell cycles driven by CRs: intermittent phase-locking of mitosis to a particular phase of the CR.

Our response: We thank reviewer 2 for highlighting the study by Charvin *et al*. This study however, did not treat coupling between CC and CR; in fact, the authors state in their first sentence that coupling between CC and CR is known to happen. Indeed, such coupling has been widely studied, both theoretically and experimentally [Yan and Goldbeter, 2019, J. R. Soc. Interface; Droin *et al.*,. 2019, Nature physics; and references therein; also recently reviewed by Chakrabarti and Michor, 2020, Curr Op Cell Biol]. The way this coupling is implemented in our model, i.e. Wee1 being a direct target of clock genes, has been experimentally established [Matsuo et al., 2003, Science] and since then, it has been used regularly in different models [e.g. Gerard and Goldbeter, 2012, PLoS CB; Yan and Goldbeter, 2019, J. R. Soc. Interface].

b. Figure 9H is strange: CR of period 12 h, 19 h or 31 h??

Our response: We agree that these values for the circadian rhythm are not biologically relevant, and could therefore be confusing. As the information in this plot was partially redundant with Figure 9I, we now removed this panel from the figure.

c. Should include a reference to Yan & Goldbeter (J R Soc Interface, 2019).

Our response: done.

Reviewer 3 comments

The manscript “A modular approach for modeling the cell cycle based on functional response curves” proposes a way to reduce the complexity of the model by replacing the detailed reaction mechanisms of modules (ultrasensitive responses, S-shaped responses and time delays) in the model. The proposed simple approach to reduce the complexity of the model is interesting and useful to investigate complex systems. I believe that the manuscript fits well to the aim of PLOS Computational Biology.

Our response: We thank reviewer 3 for the careful reading of our work, which was mentioned to be interesting and useful, as well as fitting well to the aims of PLoS Comp Biol. Here below, we address the different comments.

Major comments

1. While the manuscript focuses on illustrating how to use the approach with various examples, the validity of this approach is rarely discussed. That is, for the validation of this approach, one expects the comparison between the dynamics of models based on mass-action kinetics having one of ultrasensitive responses, S-shaped responses and time delays and their simplified models with the proposed approach. Thus, please illustrate which parts of the dynamics of the original model are quantitatively or qualitatively captured by the simplified model. Importantly, please also illustrate which part of the dynamics of the original model cannot be captured by the simplified model. This will provide a guideline for the readers to use the proposed approach.

Furthermore, from Fig. 3 to Fig. 8, various features of the systems with ultrasensitive responses, S-shaped responses and time delays are predicted with the simple models. Please describe that which predicted features match with previously known ones and which features are newly predicted. The matched features will indirectly support the validity of this approach and the new features will show the usefulness of the approach.

Our response: This is an excellent question that goes to the heart of this approach, and we thank reviewer 3 for highlighting the lack of this discussion in our work. We now discuss how fitting the S-shaped (and ultrasensitive) response curve only accurately predicts the temporal behavior of the system if time scales are well separated, such that the time traces stay close to the steady state response curves. A detailed study of all possible molecular mechanisms leading to bistability, ultrasensitivity and delay, together with validity criteria for modeling these responses in a phenomenological way, is an immense undertaking that goes beyond the scope of the present paper. Nevertheless, we follow reviewer 3 in his/her argument that such validity criteria are very important as they define the practical use of the model. Therefore, we tried to include references to earlier work in which these aspects have been treated, such as the review mentioned in the next comment and [Rombouts *et al*., 2018, Plos One].

As now added to the Discussion, the findings in this work are in line with previous work showing how ultrasensitivity, bistability and delay embedded in a negative feedback loop promote oscillatory behavior of a system. Whereas most of this earlier work has studied these different modules in isolation, we provide a straightforward method to study their combined effect. By combining delay with bistability for instance, we could show how longer delays increase the robustness of the oscillations against changes in the width of the S-shaped region, but that a maximal delay exists beyond which no additional advantage is achieved. Although the effect of combining two bistable switches on the overall bistability of the system has been studied before [Rata *et al*., 2018, Current Biology], we demonstrated the existence of two distinct ‘oscillatory regimes’ (i.e. low vs high amplitudes).

2. The proposed approach is based on Hill-functions. A recent review (Kim and Tyson, PLOS Com Biol (2020)) illustrates the risk of using such functions to describe protein-protein interactions. In particular, the key modules and examples discussed in this manuscript are usually generated by protein-protein interactions. Please discuss under which condition the proposed approach can be applicable to describe the modules generated by protein-

protein interactions and whether the approach can be extended via the function based on tQSSA rather than Hill functions.

Our response: As discussed in the previous comment, we now state the conditions under which the approach of fitting response curves can capture the dynamics of the overall system. Although we shortly mention the importance of time scale separation for approximations other than the one introduced here (e.g. the QSSA) and refer to the reference by Kim and Tyson for this, we do not explicitly discuss the use of the tQSSA. Because in our approach, we start from the steady-state response (e.g. as measured experimentally), no mass-action reaction equations are present that can be subjected to either the sQSSA or tQSSA.

3. Authors argue that asymmetric bistable APC/C nullclines slow down APC/C (in)activation. However, the dynamics author used can also be slowed down if one uses symmetric APC/C nullclines with smaller APC_{RF} value and larger APC_{LF} value. Thus, asymmetry of nullclines seems not required to obtain slow dynamics.

Our response: Reviewer 3 is indeed right in saying that other options exist to slow down APC/C (in)activation and we apologize for the confusion. In line with comments of other reviewers, this section is now removed from the text.

Minor comments

1. In Figure 6, oscillations with large amplitudes are considered as robust oscillations. Please clearly state that for what type of perturbations, the oscillator is robust

Our response: Thank you for highlighting this lack of explanation. We now added that "the oscillator becomes more robust for changes in the width of the S-shaped response curve".

2. The legend of Fig 4B mis-illustrates figures.

Our response: This figure and the corresponding section of the text were removed from the text (see also major comment 3).

3. In figure 6DE, relative synthesis rate c is not specified.

Our response: The correct value of parameter c is now included in the caption of the figure.

4. Not only for DNA damage and circadian clock coupling, but also for the circadian clock itself, the bistability plays the important role (Beesley S et al, PNAS (2020)).

Our response: This reference is now included in the text.
