# Supplementary material for: A modular approach for modeling the cell cycle based on functional response curves
Source: PLoS Comput Biol. 2021 Aug 11;17(8):e1009008. doi: 10.1371/journal.pcbi.1009008 (PMC8382204; doi:10.1371/journal.pcbi.1009008)
Supplement: Attachment — Submitted filename: Response_editor_reviewers.pdf [file pcbi.1009008.s015.pdf]

KU LEUVEN

LABORATORY OF DYNAMICS IN BIOLOGICAL SYSTEMS
HERESTRAAT 49
3000 LEUVEN, BELGIUM

Plos Computational Biology
2021-07-14

Rebuttal letter "A modular approach for modeling the cell cycle based on functional response curves"

Dear editor,

Thank you for the feedback on our manuscript entitled "A modular approach for modeling the cell cycle based on functional response curves". We are glad to read that two out of three reviewers approve publication of our work without any further changes.

Upon resubmission you asked us to focus on:

- mentioning the alternatives of a limit cycle representation of the cell cycle
- providing the codes of the models, either as supplement or on a repository
- answering the three major comments of referee 1

Below we provide an answer to the remaining questions of referee 1 (both major and minor comments). We also provide a reply to referee 2, including a short discussion about the limit cycle representation of the cell cycle as was asked for. All codes used in the manuscript were already shared on a GitHub repository in our previous revision. We believe referee 1 might have missed this as referee 2 did select "yes" to the question whether all codes were fully available.

Yours sincerely,

Lendert Gelens

LENDERT GELENS
TEL. +32 16 37 42 88
lendert.gelens@kuleuven.be
www.gelenslab.org/

Reviewer 1 comments

Reviewer #1: I have carefully read the revised manuscript, and I thank the authors for having clarified some aspects that I have raised in the first round of revision. A few explanations have been now added where required, and almost all issues raised in the previous round of review have been carefully addressed. However, despite improvements have been made, a number of technical concerns remains, mainly regarding the S-shaped module and the way in which time delays have been incorporated into the model. Specifically:

- Regarding (system (iii)) – former major concerns 2) and 3) – , I believe that confusion arising from the names of the state variables has been resolved, and I thank the authors for having clarified this point. Concerning the ODEs, I understand the reason for multiplying the RHS of the ODE by [CycB]; the explanation provided by the authors is reasonable. However, according to this reviewer, it seems to be incorrect to describe (system (iii)) as the composition of the previously introduced modules, since the RHS of the ODE describing the dynamics of [CycB] does not correspond to any of these modules.

Our response: Although the multiplication with CycB does not affect the applicability or validity of our approach, we see how referring to this equation in system iii as a module identical to the one used in system ii might be confusing. Accordingly, when discussing system iii, we now explicitly emphasize the fact that this system is based on "a slightly modified version of the S-shaped response compared with system ii".

- Regarding the computation of the derivative of the expression for the non-dimensionalized inverted bistable response curve – former major comment 4) –, I thank the authors for having inserted a detailed technical explanation (page 29, lines 941-943). However, according to this reviewer, a technical mistake exists in their reasoning. The claim "x is a function of y, and y a function of z" is only partly correct, since y is a fucntion of both x and z (i.e. [CycB] and [Cdk1], respectively). If x is a function of y and y is a function of z, then the application of the chain rule of differentiation yields, as correctly indicated by the authors, dx/dz = (dx/dy)(dy/dz). However, this seems to be not the case the authors are dealing with. The problem in their formulation (which is absolutely correct when x=[Cdk1] and y=[APC]) is that x=[CycB] is treated as an independent variable in the RHS of the equation whereas it is treated as the dependent variable in the LHS of the equation.

Our response: We see how the introduction of x, y and z may be confusing. In calculating the derivative dx/dz, we treat both x and y as functions of z so that the chain rule can be applied. We have now rewritten this part of the supplemental information to clarify this point.

- Regarding the section "Delay increases the period, amplitude and robustness of oscillations" – former substantial explanation 8) –, I acknowledge that it has been reorganized in a clearer way. However, concerns still remain regarding the temporal delays τ_1 and τ_2. Specifically, according to this reviewer, the way time delays have been incorporated into the model does not reflect the provided biological explanation. Since the temporal delay τ_1 is the delay on the "left branch" of the network in Figure 5A (i.e., form Cdk1-CycB to APC) and τ_2 is the delay on the "right branch" of the network in Figure 5A (i.e., form APC to Cdk1-CycB), I would have expected τ_1 in the second ODE and τ_2 in the first ODE. In the equations of (system (ii)), the dynamics of APC/C is alternatively affected by the "activation delay" and by the "inactivation delay". In this way, τ_2 does not reflect the meaning of this delay, which is the delay in the inactivation of CycB-Cdk1 by APC/C. Furthermore, it seems to be reasonable that, if the time delay depends on the state of the system, then this dependence exists both in case τ_1 equals τ_2 and in case τ_1 differs from τ_2. On the contrary, in the provided expression for the time delay τ, the time delay depends on the state of the system only if τ_1 and τ_2 are different. For which biological reason is this value independent of the state of the system only if the "activation delay" and the "inactivation delay" have the same value?

Our response: Some confusion appears to exist about the role of the time delays τ1 and τ2. Both delays represent the delay between changes in APC/C activity following changes in Cdk1 activity, i.e. both are situated on the left branch of the network in Fig. 5A. Whereas τ1 denotes the delay in activation of APC/C once Cdk1 crosses a thresh-old 'on its way up', τ2 denotes the delay in inactivation of APC/C after Cdk1 levels passed the left threshold 'on the way down'.

We have implemented these different activation and inactivation delays using a state-dependent delay. We use an expression for the state-dependent delays based on a Hill function which approaches a step function, so that this function represents two discrete values of the time delay, i.e. τ1 and τ2. The delay depends on APC/C activity in such a way that for low levels of APC/C, the delay is τ1 (the delay on the way up), whereas for high levels of APC/C it is τ2 (delay on the way down). In case both delays are equal, the Hill function used to model these differences in delays reduces to a constant value. We tried to clarify in the text that the goal of the state-dependent delay was to obtain two different values for τ1 and τ2.

In addition, I still have the following minor comments:

1) Regarding the difference between an ultrasensitive and an S-shaped response curve – former major concern 1) –, I understand the point made by the authors when claiming that “an ultrasensitive curve is not really S-shaped”. However, I believe that this distinction is not universally recognized as, in some cases, the terms “ultrasensitive” and “S-shaped” are considered synonyms (see, e.g., [Perelson SciTranslMed 2011], [Gunawardena PNAS 2005], [Estrada Cell 2016]). For the sake of clarity - and to avoid any possible confusion -, I would suggest to explicitly mention that by “S-shaped curve” you mean a curve “with two bumps” (or, alternatively, a more appealing description), which is hence different from an ultrasensitive curve.

Our response: Thank you for pointing out this inconsistency in nomenclature used by different authors. We now added some clarification to the abstract and the introduction when first mentioning S-shaped response: S-shaped responses should have “multiple output values for certain input values”. In this way, we hope that ‘S-shaped’ as defined in our text will not be confused with sigmoidal responses.

2) Page 13, Figure 5B (Figure 6B in the previous version of the manuscript): please, replace “bistable width” with “width of the S-shaped region” in the label of the X-axis.

Our response: done.

3) Page 13, caption of Figure 4: please, indicate the value of the constant factor by which all mass-action constants were divided.

Our response: done.

4) Page 14, lines 422-433: Please, rephrase the sentence “Such delays can be modeled in a phenomenological way by delay differential equations” with, e.g. The effects of these delays can be modeled in a phenomenological way by using delay differential equations”.

Our response: done.

5) Page 19, line 576: Regarding Figure 7B, please add also the time course of APC/C, which is currently missing.

Our response: done.

6) Page 20, line 606: please, replace “Fig. S8 Fig” with “Fig. S8”.

Our response: done.

7) Page 22, line 694: If I correctly understood, “frequencies” should be replaced with “ratio between the two frequencies”.

Our response: done.

8) Page 23, line 751: please, replace “numbers” (plural) with “number” (singular).

Our response: done.

9) Page 24, line 784: please, replace "reviewed in this journal [63]" with "reviewed in [63]".

Our response: done.

10) Page 24, line 817: "are in line with previous work". Could the authors provide a few references?

Our response: done.

11) Page 31, lines 984-985 – former previous major concern 5) – Related to the sentence "Default parameter values were manually screened so that oscillations with biologically relevant periods were obtained (Table 2), please indicate the experimental references [11] and [15] that refer to this point, as you have specifically indicated these in the response letter.

Our response: done.

Reply to reviewer 2 and the editor regarding the limit cycle representation of the cell cycle

We thank reviewer 2 for approving our manuscript for publication. We fully agree with the idea that the cell cycle is a series of irreversible transitions governed by bistable switches and that a certain drive is required to push the system across the thresholds. In our work, this drive was chosen to be part of the biochemical network itself (i.e. cyclin synthesis and degradation) and as such we obtained autonomous oscillations. The resulting limit cycle, however, is a complex one and oscillations can furthermore be arrested to account for checkpoints. In a way then, we tried to unify the vision of the cell cycle as a limit cycle 'clock' with that as a series of bistable 'dominoes'. To clarify these points, we added the following paragraphs to the discussion:

The early embryonic cell cycle can be considered as a `clock-like' oscillator, which from a dynamical systems viewpoint consists of an autonomous limit cycle oscillator [3, 99]. For more complex cell cycles, on the other hand, such as the mammalian cycle with its checkpoints, the dynamical nature is not entirely clear yet. Some authors suggest that the latter too, is a limit cycle in which the ordering of different cell cycle phases and the activity of different Cdk's naturally emerge from the underlying biochemical interaction network [101]. Another viewpoint regards the cell cycle as a series of irreversible transitions, each one generated by a bistable switch. In that case, changes in the | what we called | input variable of the switches drive the system forward and a cell cycle transition happens whenever a certain input threshold is reached. The input variable can be several things, such as mitogen concentrations, cyclin levels or cell mass [55]. Whether autonomous limit cycle oscillations can or cannot be observed, then depends on whether the dynamics of the driving force is or is not implemented in the system equations, respectively.

Our model contains elements from both the viewpoint of the cell cycle as a limit cycle and as a chain of bistable switches. The equations we used describe changes in cyclin levels, which drive the cell through the different transitions. The cell cycle emerges as an autonomous limit cycle with a fixed period. It is, however, a complicated limit cycle that conceptually differs from a simple oscillator, as the trajectory jumps between distinct states (corresponding to cell cycle phases). Moreover, our model allows for the implementation of checkpoints, i.e. temporarily pausing the spontaneous oscillations, by altering the rate constants or the underlying S-shaped responses themselves. Granted, this checkpoint action is then generated by an external input or perturbation, which makes the cycle non-autonomous. As such, our model shows features of both domino-like and clock-like behavior, and is similar in spirit to, for example, the model studied by Gonze and Goldbeter [100].
